# Developing affordable monoclonal antibodies for LMICs through high performance manufacturing processes and LMIC based commercial manufacturing

**DOI:** 10.3389/fped.2026.1880830

**Published:** 2026-09-14

**Authors:** Greg Pennypacker, Yetunde Adewunmi, Hannah Lichtenberger, Diana Herrin, Mary Jean Guillermo, Enrique Cowen, Leigh Pierce, Greg Hanson, Giovanna Chiyoko Weinman, Grace Richardson, Yosef Alemi, Nicholas J. Mantis, Tsepo Lebiletsa Tsekoa, Rachel Chikwamba, Gary Pierce

**Affiliations:** 1 ServareGMP, San Diego, CA, United States; 2 Wadsworth-Laboratory of Microbial Pathogenesis and Immunology (MPI) Division of Infectious Diseases, Wadsworth Center, Albany, NY, United States; 3 CSIR-Biomanufacturing, Council for Scientific and Industrial Research, Pretoria, South Africa

**Keywords:** cholera, continuous manufacturing (CM), LMIC (low and middle income countries), mAb—monoclonal antibody, perfusion bioreactor system, Economic Modeling

## Abstract

Monoclonal Antibodies (mAbs) are one of the most effective developments in disease prevention and treatment of the last century. Active treatment and passive immunization with mAbs targeting key antigens represents a promising approach to preventing and mitigating infectious pathogen-caused illness, particularly in Low and Middle-Income Countries (LMICs). While the development of mAbs against infectious disease has lagged behind that of immune disorders and oncology targets, recent efforts against infectious diseases have seen increasing focus with the development of mAbs against RSV, Ebola strains, and SARS-CoV-2, among others. The price for mAb treatment remains out of reach for most patients in middle income countries and nearly all patients in low-income countries. It is therefore clear that a new approach to manufacturing mAbs is needed. Here, high-productivity, high-yield mAb fed-batch production is demonstrated using clonal variants of ZAC-3, a mAb directed against the conserved core/lipid A region of *V. cholerae* lipopolysaccharide (LPS). We then extrapolate production levels seen in the ZAC-3 fed-batch run data to a continuous manufacturing approach to show a potentially viable path to manufacturing low cost mAbs at commercial scale. The reduction in cost of manufacturing drug substance resulting from the use of a continuous manufacturing process, coupled with high efficacy, and potential for low dose sizes, could give mAbs such as ZAC-3 the potential to be widely used in LMICs. The manufacture of mAbs in an LMIC-based commercial GMP facility should further reduce the cost of manufacturing, aiding in providing low-cost mAbs to patients in LMICs. Taken together, we aim to show that the potential for developing deployable antibody-based interventions to combat unaddressed pediatric diseases and rapid outbreak responses in LMICs is within reach.

## Introduction

MAbs are “clonal products defined as any intact antibody, antibody fragment, conjugate, fusion protein, bispecific, or multi-specific antibody that contains a VH -VL pair, single V domain, or combinations of single V domains where the complimentary-determining regions (CDRs) form the antigen binding site” (1). Monoclonal antibodies are laboratory generated molecules that are designed to target specific antigens (2, 3), acting through various mechanisms such as receptor blockade, antibody-dependent cellular cytotoxicity (ADCC), complement-dependent cytotoxicity (CDC), and inhibition of immune checkpoints (4, 93).

Since the approval of the first mAb in 1986 (5), mAbs have revolutionized the treatment of disease, including cancers, immune disorders, and infectious diseases (5, 95, 106, 107, 110). MAb specificity, efficacy, and versatility has created a significant track record in prophylaxis and in the treatment of a wide range of complex medical needs (6). Excluding biosimilars and combination products, 1,516 therapeutic antibodies are in clinical development worldwide and 207 registered and in commercial use (7). The US FDA approved 159 antibody-based biologics, excluding emergency use authorization (EUAs) by 2024 (8).

In 2024 therapeutic mAbs generated $252B in revenues (9), up from approximately $100B in sales in 2019 (10). Revenues are predicted to generate approximately $951.24B in revenue by 2034 (11). Revenue growth is generated from high income countries (HICs) and upper middle-income countries, and therefore the developer focus for mAbs is HIC markets. An example of this revenue sourcing is that of the recently licensed mAbs against RSV, Beyfortus. Annually, RSV causes approximately 3.6 million hospitalizations and approximately 100,000 deaths in children under 5 (116, 117). 97% of pediatric RSV caused deaths occur in LMICs (116,117). An effective, affordable mAb treatment is needed in LMICs. Yet the costs of Beyfortus preclude the needed wide usage in LMICs.

Dosing prices in LMICs for Beyfortus range between $395 and $495 (12–16). Given that the retail prices for Beyfortus are $556 for 50 mg and 100 mg doses, and $1,112 for the 200 mg dose (17) there is no significant price drop for LMICs, as would be needed to widely deploy this mAb. These prices are not affordable in LMICs, and vary greatly even within economic classifications. For example, in Argentina, a high middle-income country, a $50 dose price is suggested as affordable (15). In Vietnam, also an upper-middle income country, a price of $15 a dose was determined affordable (16). And Beyfortus is not the only mAb that is unaffordable in LMICs, as shown in Table 1.

**Table 1:** Example retail prices for various licensed mAbs from high, upper-middle, and lower-middle income countries.

County and Economic status	Trastuzumab (Herceptin)	Adalimumab (Humira)	Pembrolizumab (Keytruda)	Nirsevimab (Beyfortus)
US (High Income Country)	$152,812/12 month course (79)	$10,782.36 for 2 40 mg auto-injector pens (80)	$12,031.36 per dose every 3 weeks (81)	$556 for 50 mg and 100 mg doses, $1,112 for 200 mg dose (82)
South Africa (Upper Middle-Income Country)	$29,430.46/ 12 month course private sector (83)	$552 for prefilled carton with 2 syringes (84)	$99,862.12/ treatment cycle (85)	Pricing not yet established
Vietnam (Upper-Middle Income Country)	$ 39,062/12 month course (89)	–not widely available in Vietnam–	$2,276.26 USD (90)	–not widely available in Vietnam
India (Lower-Middle Income Country)	$3,302/12 month course (86)	–not widely available in India–	$6,495/cycle (87)	$427 per does (88)

While the deployment of mAbs has been successful in HICs, deployment in LMICs has been limited in MICs and rare in LICs (18, 19). Despite large numbers of patients who could benefit from mAbs, priorities in drug development have rarely focused on the most pressing needs in LMICs (20, 91). This reality has been known and widely remarked upon for decades (20). Deployment of mAbs is particularly limited in Africa, where 20% of the world's population resides and only 1% of mAbs sales occur (19, 104, 109). Lower MICs and LICs have seen very few mAb licensures, for example Rwanda has had 8, Uganda 5, Nigeria 10, and Zimbabwe 7 (21, 22). While factors such as regulatory hurdles and insufficient networks to efficiently distribute mAbs influence unavailability in LMICs (21), “high prices are the key barrier to accessing mAbs, with many priced at tens or hundreds of thousands of US dollars per year,” (6, 21, 23, 24, 94, 108). Examples of high prices are shown in Table 1. While the price of mAbs is different than the cost to manufacture, the price of a mAb to patients is significantly influenced by the costs to manufacture such drugs (25).

Given these realities, it is encouraging that efforts to address the lack of mAbs deployment are occurring through enabling nations in Africa to develop internal drug manufacturing capacity (26–28). However, simply transferring processes with high manufacturing Cost of Goods sold (COGs) may not result in broad availability of low cost mAbs. Therefore, significant reduction in manufacturing COGs may be needed to achieve the goal of widespread use of mAbs in LMICs.

## Section One: ZAC3 against *V. Cholerae*

### ZAC-3 introduction

With mAbs promising versatility and efficacy, they present an opportunity to combine treatment and prophylaxis, creating a two-pronged approach that could address issues caused by the many common and deadly diseases impacting LMICs. One target for a mAbs development program is the disease Cholera. An acute diarrheal infection caused by the Gram-negative bacterium *V. cholerae*, cholera has been afflicting global populations for centuries and is capable of causing death within hours in the absence of intervention (96). Since 1817, the world has experienced six pandemics, with the seventh currently affecting large swaths of Africa and Asia (29, 30). Upon entering the human digestive tract, *V. cholerae* utilizes a single polar flagellum to reach the proximal small intestine, where it penetrates the viscous mucus layer overlying the epithelium and gains access to villus crypts. Following intestinal colonization, the bacterium secretes cholera toxin (CT), an ADP-ribosylating toxin that triggers severe watery diarrhea (“rice water stool”), the hallmark of the disease and driver of mortality. Infection is typically brought on by consuming contaminated food or water (97). Infection caused illness is often more severe in malnourished individuals, further impacting prevention and treatment in LICs with more limited public health infrastructure than HICs.

While CT is an obvious target for prophylactic development, antibodies against CT are not particularly effective in limiting disease, presumably because the toxin is released from the bacterium directly onto the intestinal epithelium, with little opportunity for antibodies to interfere with toxin binding or uptake (31). While there are multiple antigens on the bacterial surface to consider as possible targets for immunotherapy, such as toxin-coregulated pilus (TCP), a compelling body of evidence from human vaccine studies and animal models indicates that protection against *V. cholerae* infection is primarily associated with antibodies against lipopolysaccharide (LPS) (32–34). Anti-LPS antibodies exert multiple effects on *V. cholerae* virulence, including motility arrest, bacterial agglutination, interference with bacterial colonization of the intestinal epithelium, and promotion of complement-mediated bactericidal (vibriocidal) activity. LPS consists of a membrane-proximal conserved core-lipid A region and a membrane-distal O-polysaccharide (OPS). The OPS of the O1 serogroup consists of (1 → 2)-linked moieties of 4-amino-4,6-dideoxy-α-D-mannopyranose (D-perosamine), in which the amino groups are acylated with 3-deoxy-L-glycero-tetronic acid. Within the O1 serogroup, there are two serotypes, Ogawa and Inaba, which differ in the presence (Ogawa) or absence (Inaba) of methylation of the terminal non-reducing D-perosaminyl moiety of OPS (35). The core/lipid A moiety is conserved between O1 serotypes and among serogroups and is therefore a broadly reactive target for cholera.

ZAC-3 is one of only a handful of known mAbs directed against the conserved core/lipid A region of *V. cholerae* LPS. Originally isolated as a mouse mAb, a chimeric ZAC-3 IgG1 antibody was generated in which the heavy (VH) and light (VL) chains were cloned onto a human IgG1 framework (36, 98). Chimeric ZAC-3 IgG was successfully expressed in a Nicotiana benthamiana-based rapid antibody manufacturing platform (RAMP). By ELISA, chimeric ZAC-3 IgG reacted with the classical Ogawa strain O395, the El Tor Inaba strain C6706, as well as the Inaba strain used in the Dukoral® cholera vaccine. We have shown repeatedly that ZAC-3 IgG, when administered to mice by gavage, significantly reduced intestinal colonization of *V. cholerae* strains O395 and C6706, revealing the potential of antibodies against the core/lipid A to contribute to immunity across biotypes (36, 37). In addition, we demonstrated that ZAC-3 arrests motility of the classical Ogawa strain O395 as well as the El Tor Inaba strain C6706, and is capable of inducing complement-dependent vibriocidal activity *in vitro*. In this study, ZAC-3 is advanced as a translational candidate for cholera prophylaxis by characterizing *in vivo* efficacy, *in vitro* functional activity.

### ZAC-3 methods and materials

#### Cell line selection

Historically, Chinese Hamster Ovary (CHO) cell lines have been the primary vehicle to produce developmental and commercially licensed mAbs. However, CHO based production has not been able to achieve the high-productivity levels needed to help reduce the price of mAbs to affordability beyond HICs (38). Novel approaches to mAb production have been proposed, and continue to be developed, including fungal and plant systems. Despite their promise, none of these technologies has achieved widespread regulatory approval allowing use for patients. Further, novel manufacturing platforms would likely require a more rigorous testing regime to achieve such regulatory approval. These obstacles limit the likelihood that alternate expression systems can be used to deploy mAbs to LMICs in the short term. Therefore, when combined with recent advances CHO based process productivity (39, 40), and widespread use of CHO expression in licensed mAbs, a CHO production process remains the most feasible option in generating mAbs deployment to patient use mAbs in the near term. As a result, for ZAC-3's development, we used a miCHO® (CHO-K1 stable) stable cell line provided by ATUM Biologics. These CHO cells were engineered to limit obstacles to effective transfection commonly seen in traditional random integration methods, allowing for more active copies of the Gene of Interest (GOI) in each cell. Cells are then able to achieve higher production than that seen in traditional CHO cell lines. After process development, these cell lines produced replicable titers of ∼9 g/L, with some runs of ZAC-3 production exceeding 10 g/L in a fed batch process.

#### Shake flask synthesis of ZAC-3 variants

A 21-day fed-batch process was developed performed in Thomson Optimum Growth® shake flasks due to their larger diameter neck, which allowed for a necessary increase in surface area for gas exchange. Utilizing an ATUM miCHO® (CHO-K1 stable) cell line, vials were thawed in Millipore EX-CELL® Advanced CHO Fed-batch Medium warmed to 37 °C and dosed with 4 mM L-Glutamine. Post-thaw, cells were placed on an orbital shaker set to 143 RPM in a CO_2_ incubator with a water pan set to 37 °C and 5% CO_2_. Cells were passaged a minimum of three times over at least two weeks utilizing un-supplemented Millipore EX-CELL® Advanced CHO Fed-batch Medium prior to being grown to the necessary volume required for final inoculation. Final production flasks were inoculated at a target Viable Cell Density (VCD) of 7.5 × 10^5^ cells/mL, in Millipore EX-CELL® Advanced CHO Fed-batch Medium warmed to 37 °C and dosed with 4 mM L-Glutamine and 0.5% HyClone™ Cell Boost™ 7b. On day three, a glucose reading was taken and cultures were supplemented as necessary to ensure 4 g/L concentration. Cells were allowed to grow for four days total after inoculation, or until they reached a VCD of 1.0 × 10^7^ cells/mL, where a temperature and agitation shift were performed, decreasing the temperature setting to 32 °C and increasing the agitation to 173 RPM. This adjustment acted to largely halt cell growth and increase mAb production. Throughout the remainder of the 21-day process, samples were run daily on a NOVA Stat Profile Prime, and CO_2_ was adjusted as needed to ensure the pH fell within the range 6.8–7.5. In addition, pH continued to be maintained within the target range by adjusting CO_2_ levels in the incubator, and the flasks were periodically fed. Different optimal feed strategies were developed dependent on the clone being grown. As the working volume increased with feeds, bleeds once a week were utilized to maintain a stable working volume. The LA variant was fed with 5% of initial working volume HyClone™ Cell Boost™ 7a Supplement and 0.5% HyClone™ Cell Boost™ 7b on days 4, 7, 10, 12, 14, 17, and 19. The LS variant was fed with an increasing percentage of both HyClone™ Cell Boost™ 7a Supplement and HyClone™ Cell Boost™ 7b as the study progressed.

The percentage of each feed and its corresponding addition day are as follows:

Day 4- 6.5% Cell Boost™ 7a and 0.65% Cell Boost™ 7b
Day 7- 8.0% Cell Boost™ 7a and 0.8% Cell Boost™ 7b
Day 10- 9.5% Cell Boost™ 7a and 0.95% Cell Boost™ 7b
Day 12- 11.0% Cell Boost™ 7a and 01.10% Cell Boost™ 7b
Day 14- 12.5% Cell Boost™ 7a and 1.25% Cell Boost™ 7b
Day 17- 14.0% Cell Boost™ 7a and 1.40% Cell Boost™ 7b
Day 19- 15.5% Cell Boost™ 7a and 1.55% Cell Boost™ 7b

Glucose levels were regulated as follows; on Day 3, a bolus of 4 g/L of 450 g/L glucose was added. On Day 4, glucose was added to bring the concentration to 11 g/L. This was calculated using the current glucose level and working culture volume, shown in the equation below:

$$Day\;4\;Amount\;of\;glucose\;to\;feed=\frac{\left(\left(11\frac{g}{L}-measured\;level\frac{g}{L}\right)\times culture\;volume\;mL\right)}{GLucose\;stock\;concentration\frac{g}{L}}$$

On remaining feed days, the following formulas were utilized to calculate the amount of glucose to be added to match the consumption rate until the next feed day.

$$\begin{array}{rl} & Day\;7-19\;Final\;glucose\left(\frac{g}{L}\right)\\ & =\left(glucose\;consumed\;per\;day\frac{g}{L}\times \#\;of\;days\;before\;next\;feed\right)+4\frac{g}{L} \end{array}$$

$$Day\;7-19\;Amount\;of\;glucose\;to\;feed=\;\frac{\left(\left(Final\;Glucose\frac{g}{L}-measured\;glucose\;level\;\frac{g}{L}\right)\times Culture\;Volume\;mL\right)}{GLucose\;stock\;concentration\frac{g}{L}}$$

On day 21, the harvest material was sampled, centrifuged, and processed via depth filtration before progressing to downstream purification.

#### Stirred tank synthesis of ZAC-3 variants

A 21-day fed-batch process was developed, being performed in a Distek BiOne1250® system, which mirrored the above shake flask procedures in every aspect except those listed below. The cells were inoculated to a 1.3 L initial working volume at a density of 7.5 × 10^5^ cells/mL in a 2 L BIOne Single-Use Bioreactor equipped with a drilled hole sparger and single impeller. The bioreactor process followed the same LA variant Fed-batch feed strategy as the flask process, however with an addition of 0.1% Gibco™ Poloxamer 188 Non-ionic Surfactant as a surfactant to reduce sheer forces. pH was maintained at 7.1 ± 0.1 via a pH cascade utilizing sparged CO_2_ from 0 to 80 sccm as an acid and 0.5 M NaOH as a base titrant. Dissolved Oxygen (DO) was maintained at 55% via constant sparged air at 112.5 sccm and a DO cascade utilizing O2 ranging from 0 to 495 sccm. Additionally, a constant Air overlay was maintained at a setpoint of 120 sccm. The temperature was initially set at 37 °C and the impeller speed to 290 RPM. Once the cells reached a VCD of 1 × 10^7^ c/mL, the temperature was reduced to 32 °C and the impeller speed was increased to 315 RPM. Gibco FoamAway™ Irradiated AOF Antifoaming Agent was added to the bioreactor as needed to mitigate foam throughout the process.

#### Titer analysis

Throughout the production process, purified ZAC-3 samples were analyzed utilizing absorbance data. To first purify the material, 10 mL columns were packed with 2 mL JSR Amsphere™ A3 protein A resin and equilibrated with 2.5 column volumes of 1× PBS. Samples were diluted 1:1 with 1× PBS prior to loading. After loading, the columns were washed with an additional 2.5 column volumes of 1× PBS. Elution was performed by adding 3 column volumes of 2.4 mM Sodium Acetate pH 3.5. The collected elution was neutralized to pH 7.2 with 1 M Tris-HCL pH 9.1. A 1:10 dilution of neutralized sample in 1× PBS was formulated and analyzed on the spectrophotometer at A280. The measured absorbance (Abs) was then plugged into the formulas below to calculate titer.

$$Elution=\frac{Abs*DF}{\in }\;Titer=\frac{Elution\;*\;volume\;of\;elution\;flow\;through}{volume\;of\;original\;sample}$$

#### Downstream purification

Once the 21-day upstream process was completed, material was harvested and filtered with a 0.2 μm depth filter prior to downstream purification. The downstream process consists of a capture step, using Protein A resin, viral inactivation, two ion exchange polishing steps using High S and High Q resins, viral filtration, and finally ultrafiltration/diafiltration TFF. Protein A chromatography capture was performed using JSR's Amsphere A3 Protein A resin packed into a Biorad chromatography column. The column was equilibrated with 5 Column Volumes (CVs) 1× PBS, pH 7.4 buffer. After sample application (load), the column was washed with 5 CV's of 1× PBS, pH 7.4. After washing, the protein was eluted with 3 CV's of 2.41 mM Sodium Acetate pH 3.5 and collected. Viral inactivation was performed on the elution material from the Protein A column by performing a pH adjustment using 6.0 M HCl until a desired pH of 3.0–3.1 was achieved. After the pH was successfully lowered, the sample was held at this step for 30–45 min in order to ensure complete viral inactivation. The sample pool was then neutralized to a pH range of 6.95–7.05 using 1 M Tris-HCl, pH 9.1.

Two polishing steps were then performed: one anionic exchange (AEX) and one cationic exchange (CEX). The AEX process was designed to run as a flowthrough step using Biorad's Macro-Prep High Q resin. This step worked to capture free nucleotides such as DNA and RNA, while letting the Zac3 mAb pass through into the flowthrough. 5 CVs of 20 mM Tris-HCl pH 8.2 was utilized as the equilibration buffer, and 5 CVs of 20 mM Tris-HCl, 500 mM NaCl, pH 8.2 was utilized as the elution/strip buffer. Flowthrough from the AEX step was then loaded onto a Biorad chromatography column containing Biorad's Macro-Prep High S resin and a bind and elute CEX step was performed. This step in the process was designed to remove High Molecular Weight (HMW) aggregates from the Zac3 mAb. The equilibration buffer implemented was 5 CVs of 20 mM Sodium Acetate, pH 5.4 and the elution buffer was 5 CVs of 20 mM Sodium Acetate, 500 mM NaCl, pH 5.4. During loading of the column, the target mAb binds to the high S resin. This is then washed using the equilibration buffer, and subsequently eluted via gradient elution using the buffers 20 mM Sodium Acetate, pH 5.4 as the “A Buffer” and 20 mM Sodium Acetate, 500 mM NaCl, pH 5.4 as the “B Buffer.”

After each Chromatography step, columns were stripped and cleaned utilizing 5 CVs each of 0.1 M NaOH and 20% EtOH sequentially. A Viral Filtration step was performed using a filter train assembly that incorporated a Viresolve Pro Modus 1.2 filter in series with a Millistak + Pro Pod Depth Filter 0.027 m^2^. After flushing and equilibrating the assembly, the elution pool from the previous step was pumped through the filter train at a rate of 15 mL/min until all material has been processed. To complete the downstream purification process, the material was ultrafiltered and diafiltered utilizing a TFF hollowfiber filter to remove the salts present from the polishing chromatography steps, concentrate the product to desired levels, and buffer exchange the material into the final fill or storage buffer.

### Downstream analytics

#### Measuring residual DNA

In-process analytic checks were performed on a NanoDrop 1,000 Spectrophotometer using known samples of purified plasmids and commercially purchased purified BSA standards as controls. In DNA and RNA processing, A260/A280 ratios provide quantitative insight into the purity of the sample being processed. Purified RNA is associated with an approximate ratio of 2.0, purified DNA has a ratio around 1.8 (41), and a sample of pure protein has a ratio of approximately 0.6 (42). ZAC-3 Harvest sample was purified via Protein A affinity chromatography and then loaded onto a Nanodrop to measure necessary absorbance levels.

As seen in Supplementary Table 1, the A280/A260 ratios were observed for Pre- and Post- Protein A purification and compared to Pure DNA and Pure BSA standards. To observe the reduction in A280/A260 ratios for Pure DNA with protein contamination, and increase of A280/A260 ratios for Protein with DNA contamination, controls of known amounts of Protein and DNA were formulated to confirm A280/A260 ratio trends of control samples with residual contaminants.

#### SDS-PAGE of ZAC3 HCP removal in Protein A purification.

An additional function of Protein A purification, is the ability to resolve Host Cell Proteins (HCP). To verify HCP removal, harvested ZAC3 material was run under Protein A affinity chromatography. Samples from the Column Load/Harvest, Flowthrough (FT), Washes, and Elution were run on a 4%–12% Novex™ Tris-Glycine Protein Gel as seen in Supplementary Figure 1.

#### SDS-PAGE of ZAC3 HMW aggregate removal in cationic exchange bind and elute

Cationic Exchange (CEX) is performed as a downstream polishing step to remove contaminants Protein A purification does not remove. The CEX operates as a bind and elute chromatography column, which binds ZAC3 and its HMW aggregates, and separates them based on molecular charges correlating to Isoelectric points (PI) and pH conditions (43). This data is shown in Supplementary Figure 2.

#### Bacterial strains, ZAC-3 IgG variants and growth conditions

*Vibrio cholerae* strains C6706 (El Tor) and O395 (Classical) were used in the assay development experiments. Bacterial cultures were grown in Luria-Bertani (LB) broth at 37 °C with shaking at 220 rpm for liquid culture, or on LB agar plates for solid culture. The following IgG1 variants were utilized: wild type (WT), L234A/L235A (LA), and L234S/L235S (LS). All bacterial strains and antibodies were handled using aseptic techniques and diluted down to desired concentrations for downstream applications.

### Assays for functional testing of ZAC-3 antibodies

#### Whole-cell vibrio ELISA

The effect of ZAC-3 IgG mAb from LA and LS bulk transfected clones or cell lines respectively—were initially tested for their ability to bind to viable strains of *V. cholerae in-vitro*. The protocol utilized was previously described in Doering et al. (44). Briefly, bacterial cultures were diluted to OD600 of 0.1 and aliquots were dispensed into wells of a 96 well ImmulonTM 4HBX microtiter plate. Plates were incubated by refrigerating overnight at +4⁰C and washed (0.1% PBS-Tween) to remove unbound cells. Each well in the plate was blocked with block buffer (2% goat serum in 0.1% PBS-T) for 2 h followed by the addition of serially diluted samples of ZAC-3 IgG mAb at a final volume of 100 µL. Following a 1 h incubation at room temperature, plates were washed 3X, and 100 µL of 1:2,000 goat anti-human IgG (Southern Biotech) was added to the plates. After a final wash step, plates were developed by adding 100 µL of SureBlue TMB (KPL) for 3 min followed by 100 µL stop solution (1 M phosphoric acid). Plates were read at OD450 using SpectraMax iD3 spectrophotometer with Softmax Pro 7.1.0 software (Molecular Devices) and downstream data visualization and analysis was conducted with GraphPad Prism.

#### Vibrio macroagglutination assay

Using the “snowglobe” assay developed by Lindberg et al., the ability of ZAC-3 IgG variants to promote macroagglutination in *V. cholerae* was tested (45). Overnight cultures of *V. cholerae* were subcultured in LB broth until mid-log phase. Bacterial cells were washed twice with sterile PBS (pH 7.4) by centrifugation at 6,000 × g for 5 min. Cells were then adjusted to a final concentration of∼1 × 10^8^ Colony Forming Units/mL (CFUs/mL) in PBS supplemented with ZAC-3 IgG at 10 µg/mL for a final volume of 5 mL and incubated static at room temperature for 4 h. Macroagglutination was recorded using an iPhone 6 s (Apple, Cupertino, CA) with the “Timestamp Camera” application.

#### Complement dependent vibriocidal assay

The vibriocidal activity of ZAC-3 was assessed by a protocol previously described by Doering et al. (44). Serial dilutions of pre-diluted ZAC-3 IgG whereas mixed with mid-log phase cultures of *V. cholerae* C6706 at a concentration of 4 × 10^5^ CFUs/mL supplemented with 10% guinea pig complement. Samples were incubated at 37 °C for 1 h and plated on LB plates to determine the minimal inhibitory concentration of ZAC-3 required to inactivate *V. cholerae*.

#### Bi-layer interferometry assay

The binding kinetics between ZAC-3 IgG and the biotinylated human FcRn were determined using biolayer interferometry (BLI) on the Octet system (ForteBio). All reagents and dilutions were prepared in phosphate-buffered saline supplemented with 2% bovine serum albumin (PBS/BSA), which served as the assay buffer throughout. Biotinylated human FcRn was immobilized on streptavidin SA biosensors at a concentration of 1.5 µg/mL (a concentration that was predetermined to be optimal for analyte binding and prevent biosensor overloading). ZAC-3 IgG LA/LS variants (analytes) were prepared in a two-fold serial dilution ranging from 200 to 3.125 µg/mL. The assay workflow, configured in the Octet Data Acquisition software, included a 5 min hydration and equilibration of both the biosensors and the assay plate on the instrument. The BLI assay sequence comprised a 5 min baseline in buffer, 5 min ligand loading, a second 5 min baseline, followed by 5-minute association and 5 min dissociation steps for each analyte. Due to the pH sensitivity of FcRn, regeneration using low-pH glycine buffers was omitted. Instead, separate biosensors were used for each mAb variant to preserve ligand integrity. Data was processed using the Octet Analysis software, applying reference subtraction and baseline correction. Binding kinetics, including the association rate constant (k_a_), dissociation rate constant (k), and equilibrium dissociation constant (K), were then derived.

#### Neonate mouse intragastric challenge assay

Following all *in-vitro* assays, the neonatal mouse intragastric challenge was used to confirm the functionality of ZAC-3 IgG (LS C1566) against *V. cholerae* C6706 *in-vivo*. An overnight mid-log phase culture of *V. cholerae* C6706 was washed twice with PBS by centrifugation at 6,000 × g for 5 min. ZAC3-IgG was then mixed at 3, 10 and 30 µg/mL with cultures at 10^8^ CFUs/mL and incubated mixture for 1 h at room temperature. 3–7-day old Swiss Webster pups (*n* = 10) were orally gavaged with 50 µL of solution containing bacteria ± antibody previously diluted in PBS using 24 gauge feeding needles (Harvard Apparatus). Following challenge, pups were placed in a clean cage with bedding from their home cage and nesting material and set in a humidified incubator set to 30 °C for 24 h. Pups were then euthanized at 24 h post-challenge and the small intestines were collected for CFU determination by spread plating.

### ZAC-3 results

#### Shake flask synthesis of ZAC-3 IgG variants

ZAC-3 IgG production was measured using titer absorbance data. Following the outlined production method, the ZAC-3 LA variant produced an average titer of 8.2 g/L with a standard deviation of ±0.55 over the course of ten total runs. The ZAC-3 LS variant produced an average titer of 9.6 g/L with a standard deviation of ±0.57 over the course of six total runs. The maximum viable cell density (VCD) the LA variant reached was 2.37 × 10^7^ cells/mL with a range of 1.96 × 10^7^–2.37 × 10^7^ cells/mL, averaging a peak VCD of 2.19 × 10^7^ cells/mL. While the LS variant reached a maximum VCD of 1.96 × 10^7^ cells/mL with a range of 1.44 × 10^7^–1.96 × 10^7^ cells/mL, averaging a peak VCD of 1.75 × 10^7^ cells/mL.

#### Stir tank synthesis of ZAC-3 IgG variants

ZAC-3 IgG stir tank production was measured using titer absorbance data. Following the outlined bioreactor production method, the ZAC-3 LA variant produced an average titer of 7.7 g/L with a standard deviation of ±0.1 over the course of two total runs. This variant reached a maximum VCD of 1.76 × 10^7^ cells/mL. Upcoming studies will further the development of the LA variant and utilize the LS variant to explore the comparability between the two cell lines and further optimize this method in a stir tank bioreactor.

#### Measuring residual DNA

The A260/A280 ratios observed across the data in Supplementary Table 1 demonstrated that Protein A is effective at removing residual DNA from the harvest material. The Pure Plasmid samples worked effectively as controls, producing ratios of 1.84. ZAC3 harvest samples showed lower levels of DNA than those seen in the plasmid samples, but were still significantly high enough to confirm the presence of residual DNA in the load material, prior to Protein A purification. Elution samples from the Protein A run both produced an absorbance ratio of ≤0.55, indicating that residual DNA was no longer present in detectable levels within the eluate.

#### SDS-PAGE of ZAC3 HCP removal in Protein A purification

As seen in Supplementary Figure 1, the bands representing HCP in the initial load material, prior to purification were significantly removed by the Protein A process. This indicates that there is no undesired binding of HCP occurring during the purification, and it is operating as intended.

#### SDS-PAGE of ZAC3 HMW aggregate removal in cationic exchange bind and elute

As shown in Supplementary Figure 2, ZAC-3 was successfully able to bind to the CEX column, and further contaminants such as HMW aggregates can be seen in the high conductivity strip, showing separation of the HMW aggregates from the ZAC3 sample and demonstrating that the CEX purification step is functioning as intended.

#### ZAC3 IgG variants bind and agglutinate classical and El Tor *V. cholerae* strains

To evaluate the binding activity of ZAC-3 IgG variants, ELISAs were performed against two *Vibrio cholerae* strains representing the classical (O395) and El Tor (C6706) biotypes. All three ZAC3 variants—LA, LS, and WT—exhibited strong binding to strain O395 across a wide concentration range, with similar OD_450_ values observed among the variants as seen in Figure 1A. In comparison, Figure 1B demonstrates binding to strain C6706 was slightly reduced but remained detectable across all variants. The differences in ELISA signal intensity were modest and may reflect strain-specific differences in surface antigen presentation, accessibility, or other contextual factors rather than epitope divergence, as the ZAC-3 target is known to be conserved between strains. Macroagglutination assays were performed with ZAC-3 premixed with *V. cholerae* and recorded time-lapse videos. The results, as seen in Figures 1C,D, showed that all ZAC-3 variants induced rapid and robust macroagglutination in both O395 and C6706 strains, as evidenced by the visible clearing of the supernatant and pellet formation in tubes containing LA, LS, and WT antibodies. In contrast, the isotype control (IC) showed no visible agglutination in either strain. These results demonstrate that despite minor differences in ELISA signal, ZAC3 IgG variants are capable of mediating effective agglutination of both classical and El Tor *V. cholerae* biotypes.

**Figure 1:**
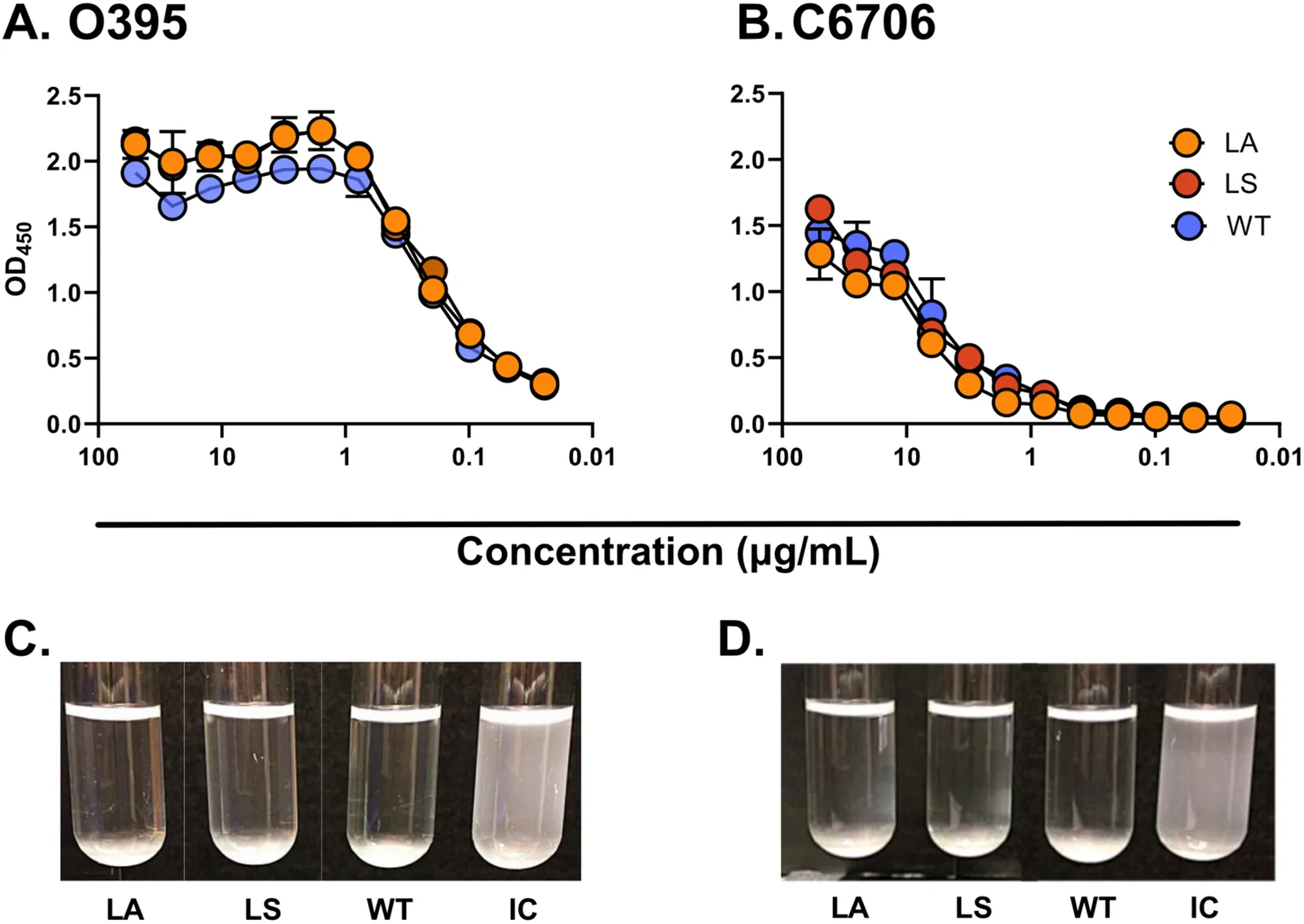
Functional activity of ZAC-3 IgG variants against *V. cholerae*. **(A)** Binding of ZAC-3 IgG LA, LS, and WT variants to the classical *V. cholerae* O395 strain assessed by whole-cell ELISA. **(B)** Binding of ZAC-3 IgG LA, LS, and WT variants to the El Tor *V. cholerae* C6706 strain assessed by whole-cell ELISA. **(C)** Macroagglutination activity of ZAC-3 IgG LA, LS, and WT variants against *V. cholerae* O395, demonstrated by visible clearing of the bacterial suspension and pellet formation compared with the isotype control. **(D)** Macroagglutination activity of ZAC-3 IgG LA, LS, and WT variants against *V. cholerae* C6706, similarly demonstrated by visible clearing of the bacterial suspension and pellet formation compared with the isotype control.

#### Functional characterization of LA and LS cell clones of ZAC-3 IgG1

The antibody binding, complement-dependent killing and bacterial macroagglutination activity of ZAC-3 IgG1 LA or LS cell clones, and their respective pools against *V. cholerae* C6706 were determined. The ELISA results show that both LA (C458 and C1001) and LS (C0250 and C1566) cell clones exhibited dose-dependent binding to C6706, with binding capacities comparable to those of their respective cell pools (LAP16 and LSP18) (Figures 2A, C). All cell clones showed clear and robust binding to the target strain with no notable differences in binding observed between samples, confirming successful antigen recognition by the antibodies, as shown in Figures 2A,C.

**Figure 2:**
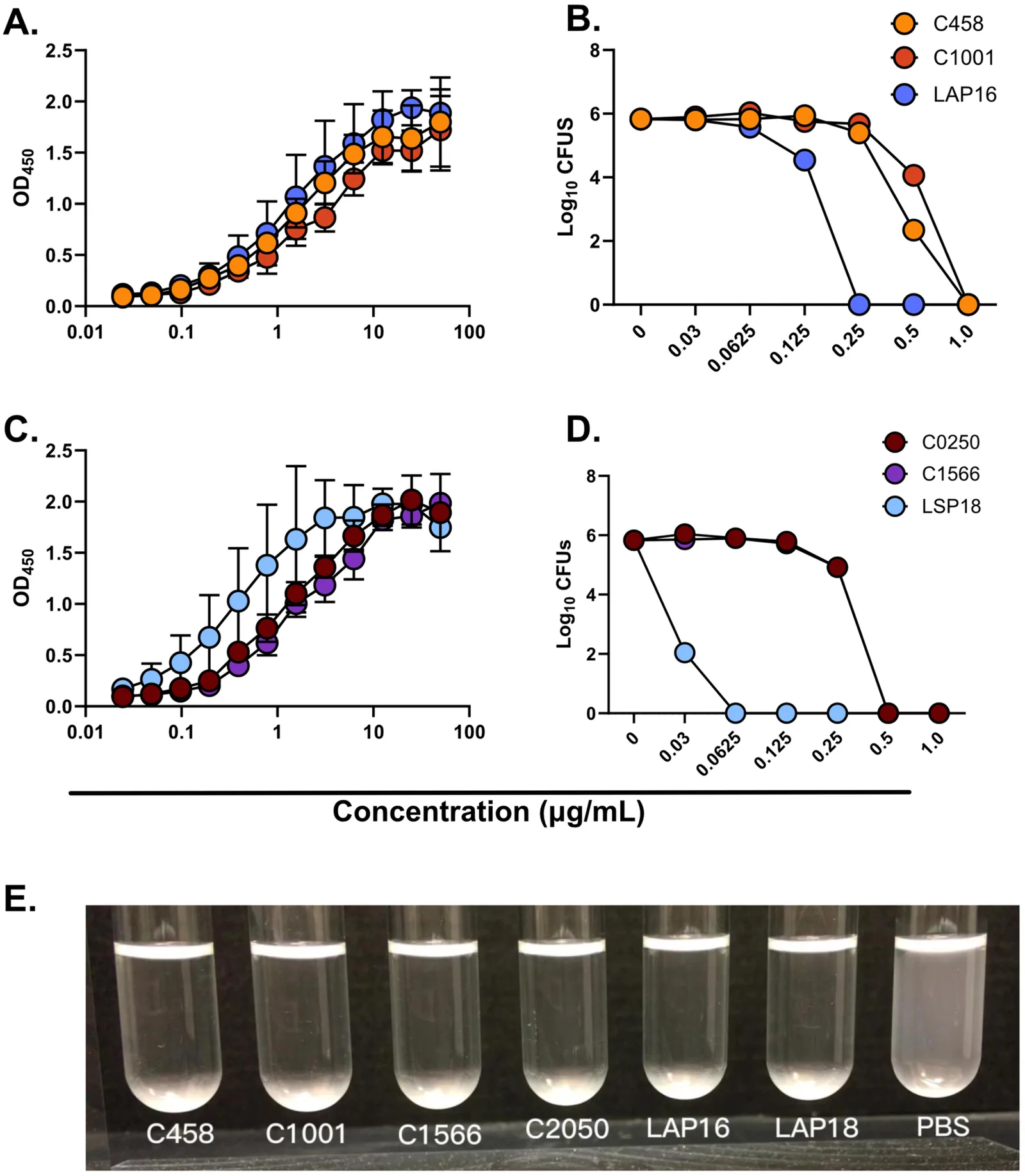
Functional characterization of ZAC-3 LA and LS clones. **(A)** Binding of ZAC-3 LA clones to *V. cholerae* C6706 assessed by whole-cell ELISA using two-fold serial dilutions beginning at 50 µg/mL. **(B)** Complement-dependent vibriocidal activity of ZAC-3 LA clones against *V. cholerae* C6706 assessed at 1:2 serial dilutions from 1.0 to 0.03 µg/mL, with 0 µg/mL included as the antibody-free control. Bacterial survival is expressed as log₁₀ CFUs. **(C)** Binding of ZAC-3 LS clones to *V. cholerae* C6706 assessed by whole-cell ELISA using two-fold serial dilutions beginning at 50 µg/mL. **(D)** Complement-dependent vibriocidal activity of ZAC-3 LS clones against *V. cholerae* C6706 assessed at 1:2 serial dilutions from 1.0 to 0.03 µg/mL, with 0 µg/mL included as the antibody-free control. Bacterial survival is expressed as log₁₀ CFUs. **(E)** Macroagglutination activity of ZAC-3 LA and LS clones against *V. cholerae* C6706. ZAC-3 antibodies were premixed with *V. cholerae* at a final antibody concentration of 10 µg/mL and assessed for visible bacterial agglutination. Macroagglutination was indicated by clearing of the bacterial suspension with formation of a visible pellet, whereas the PBS control remained turbid.

Complement-dependent vibriocidal assays further revealed significant functional activity among the cell clones. Specifically, the data in Figure 2B demonstrates that both LA clones C458 and C1001 achieved complete vibriocidal activity at 1 µg/mL; however, at 0.5 µg/mL, C458 reduced bacterial counts by nearly 100-fold more than C1001, demonstrating enhanced killing efficiency at lower concentrations. Also seen in Figure 2B, LAP16 pool exhibited the highest bactericidal activity, completely eliminating viable bacteria (6-log fold) at 0.25 µg/mL. In general, the LS ZAC-3 IgG had higher vibriocidal activity compared to their LA counterparts as seen in Figure 2D. Also seen in Figure 2D, the LS clones impaired the survival of *V. cholerae* C6706 at 0.5 µg/mL with the LSP18 pool achieving vibriocidal activity at 0.0625 µg/mL.

#### ZAC-3 IgG1 reduces *V. cholerae* Burden in Mouse Intestinal Tissues

The functionality of ZAC-3 IgG LS C1566 against *V. cholerae* C6706 *in-vivo* was confirmed using a neonate mouse model. Briefly, ZAC-3 C1566 was preincubated at varying concentrations with *V. cholerae* C6706 1 h before oral gavage to allow for antigen-antibody binding as shown in Figure 3A. 24 h following oral gavage with antigen-antibody complex, results showed significant reduction in the burden of *V. cholerae* C6706 in the gut tissues of pups in a concentration dependent manner with Figure 3B showing the most significant drop occurring at 30 µg/mL compared to the isotype control. These results establish the *in-vivo* functionality of ZAC-3 IgG1 C1566, highlighting its potential as an effective antibody against *V. cholerae* intestinal colonization.

**Figure 3:**
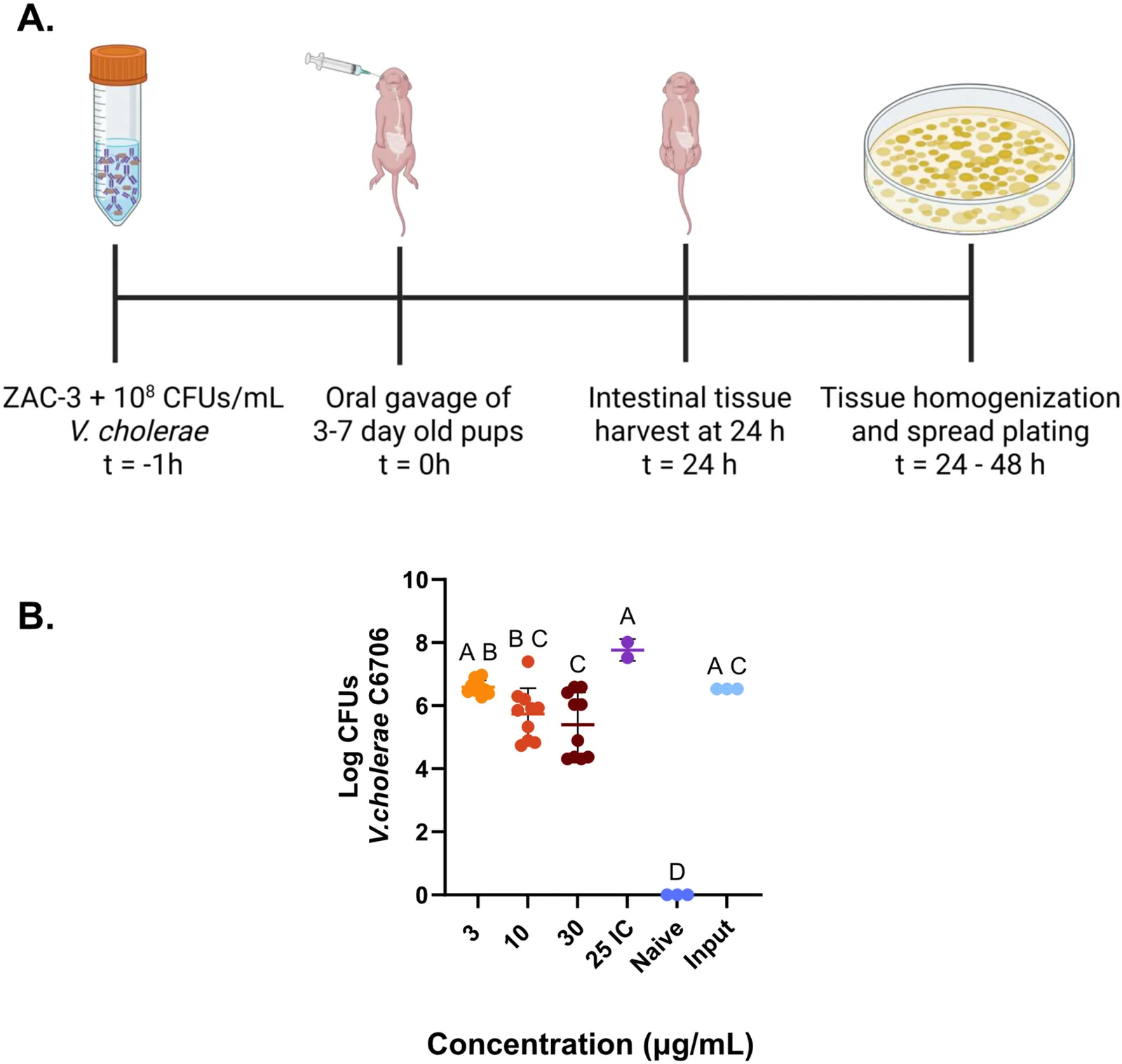
Functional activity of ZAC-3 C1566 *in vivo* via mouse neonatal challenge. **(A)** Schematic depicting the neonatal mouse challenge in Swiss Webster mice. ZAC-3 IgG was preincubated with *V. cholerae* C6706 prior to oral gavage of neonatal pups, followed by collection of intestinal tissues 24 h post-challenge, tissue homogenization, and plating for determination of bacterial burden. **(B)** Bacterial burden in the intestinal tissues of pups was quantified 24 h post-challenge, demonstrating a concentration-dependent reduction in *V. cholerae* C6706 colonization with increasing ZAC-3 C1566 concentrations.

#### ZAC-3 IgG1 interacts with FcRN

Biolayer interferometry revealed that all Zac3 IgG variants, highlighted in Table 2, had high-affinity interactions with human FcRn in the nanomolar range. The equilibrium dissociation constants (K_D_) ranged from 0.56 to 0.85 nM, indicating uniformly high-affinity interactions across both LA and LS variants. Notably, LAP16 (LA) exhibited the strongest binding (K_D_ = 0.56 ± 0.04 nM), followed closely by LSP18 (LS) at 0.65 ± 0.04 nM. These tight interactions were primarily driven by low dissociation rates, suggesting that both Fc-engineered pools and individual clones maintain favorable FcRn engagement. The consistency across clones highlights that Fc engineering did not compromise FcRn binding, and in some cases subtly enhanced affinity compared to wild-type IgG benchmarks.

**Table 2:** ZAC-3 IgG1 human FcRN binding by clone.

Variant	Isotype	KD (nM)
C458	LA	0.62 ± 0.01
C1001	LA	0.82 ± 0.02
LAP16	LA	0.56 ± 0.04
C1566	LS	0.85 ± 0.02
C2050	LS	0.76 ± 0.02
LSP18	LS	0.65 ± 0.04

### ZAC-3 discussion

The provided data advances ZAC-3, originally isolated as an IgA from mouse Peyer's patch B cells and subsequently expressed on a human IgG1 backbone, as a candidate monoclonal antibody for translational use. By engineering ZAC-3 IgG variants suitable for large-scale manufacture and extended half-life, these data extend prior studies that have shown its ability to block motility and colonization, while positioning this antibody for outbreak response settings where rapid protection is essential. Taken together, the literature and our studies support three actionable implications. First, antibody-based prophylactics could fill an important gap in outbreak response, especially for young children who are ineligible or respond poorly to polysaccharide-based vaccines, by providing immediate, biologically plausible protection that acts at several steps in pathogenesis. Second, the LPS-directed mechanism of action means ZAC-3 and similar monoclonal antibodies can be expected to exert multiple barriers to colonization (motility arrest, agglutination, complement activation), which is advantageous for preventing both disease and transmission. Third, to realize population-level impact, the antibody must be manufacturable and stable under field conditions, goals that require continued investment in low-cost manufacturing platforms and thermostable formulations.

The production data, generated from the outlined fed-batch runs in CHO-K1 cell lines, shows strides towards achieving the high titers needed for financial relevance in LMICs. These results reaffirm the ability of modern CHO cell lines to produce titers approaching, at, or above 10 g/L, surpassing historical yields, and providing a potential opportunity to exploit this high production level in more continuous manufacturing processes.

The presented data also supports the premise that antibodies which bind LPS can mediate multiple non-redundant mechanisms that reduce intestinal colonization and disease severity, including rapid motility arrest, bacterial agglutination, interference with mucosal attachment—which prevents the production and release of cholera toxin, the exotoxin responsible for the severe diarrheal symptoms of cholera—and complement activation for bactericidal activity. These multimodal effects explain why LPS-directed antibodies can show potent protection that is not captured by a single *in-vitro* assay. The concept of blocking motility and promoting agglutination as front-line barriers to colonization is particularly relevant for prophylactic use, since antibodies that prevent initial niche establishment will lower both the risk of symptomatic disease and onward transmission.

### ZAC-3 conclusion

LPS-targeting monoclonal antibodies—such as the engineered ZAC-3 IgG variants described here—represent a biologically rational and operationally promising strategy to provide rapid, targeted protection against cholera, with particular potential to protect young children and to be used in outbreak emergency response if the manufacturing and formulation strategies discussed here can be scaled appropriately.

## Section Two: Modeling continuous manufacturing

### Introduction

MAbs have traditionally been very expensive to manufacture for two primary reasons, complexity of the manufacturing process and the high cost of facilities, materials, and components. This resulted in very high prices for mAb doses. Between 1997 and 2016 price per gram ranged from $4,650 per gram to $114,318,850 per gram (6, 25). The complexity of manufacturing processes and their low productivity created the need for large, costly facilities, which required large capital expenditures to establish and operate. Further, the high cost of materials and components also contributed to the prohibitive costs of mAbs for use in LMICs. Low levels of cell line productivity at ≤ 1 gram per liter of culture have also contributed, requiring very large production runs scales and bioreactor vessels to generate sufficient mAbs for patient dosing, further exacerbating mAb pricing. More recent commercial products, such as Regeneron's mAb against SARS-CoV-2 (92), while showing reductions from the earlier cost range, with manufacturing cost of approximately $2,000 a gram (25, 46), are not significant enough to achieve manufacturing costs low enough to be relevant in LMICs. However, the industry continues to see reductions in COGs, dropping from multiple thousands of dollars per gram to the hundreds and even less than $100 per gram (47). Improvements in cell line expression, fed batch production, and purification processes have been drivers for manufacturing cost reductions. Further, the integration of single use processing methods likely contributed to cost reductions as well, as single use processes are substantially more cost-effective to utilize than traditional reusable stainless-steel systems (12, 48, 49, 102, 103, 111, 115). While impressive. these developments have not facilitated widespread distribution of low cost mAbs into LMICs.

As shown in the Scenario Based Economic Model section below and in Supplemental Materials, even a high-performance fed-batch process will not bring COGs to manufacture DS down to a level that would allow for affordably priced mAbs in LICs, and likely not lower-level MICs either. In this section we explain how a continuous manufacturing process, adapted from the described fed-batch process used to manufacture ZAC-3, can potentially reduce COGs down to a level that would assist in deploying very low price mAbs in LMICs.

With alternatives to traditional batch processes, COGs for mAb production and purification can be reduced compared to COGs for batch (50) and even fed-batch manufacturing processes. Through process optimization methods such as n-1 intensification, continuous perfusion production, aggregate reduction via optimized pH elution profiles, and increasing binding optimization utilizing multi-column chromatography in downstream, the opportunity exists to make cost-effective mAb DS. These methods can enhance efficiency, improve production output, and accelerate operating time, potentially reducing costs enough to increase access to mAbs through lower prices (101).

Conventional upstream fed-batch processes, seen in Figure 4, are inoculated at lower cell densities, thus extending the time it takes to reach the target maximum cell density for production (51). This increases manufacturing costs to obtain the desired titer results. Achieving higher titers at a faster rate and increasing the stability of the cell culture will lower overall manufacturing costs. N-1 intensification is an expansion method where the last step of the seed train process is concentrated to a high cell density (52, 53, 99). This higher cell density is used to inoculate the production run at an increased cell density compared to most conventional processes (54). The higher initial concentration shortens the time it takes to reach the maximum VCD, increasing the titer output, and reducing the facility space needed to scale up for a production run (54). All are factors that contribute to a reduction in COGs for the overall process (50, 55, 56).

**Figure 4:**
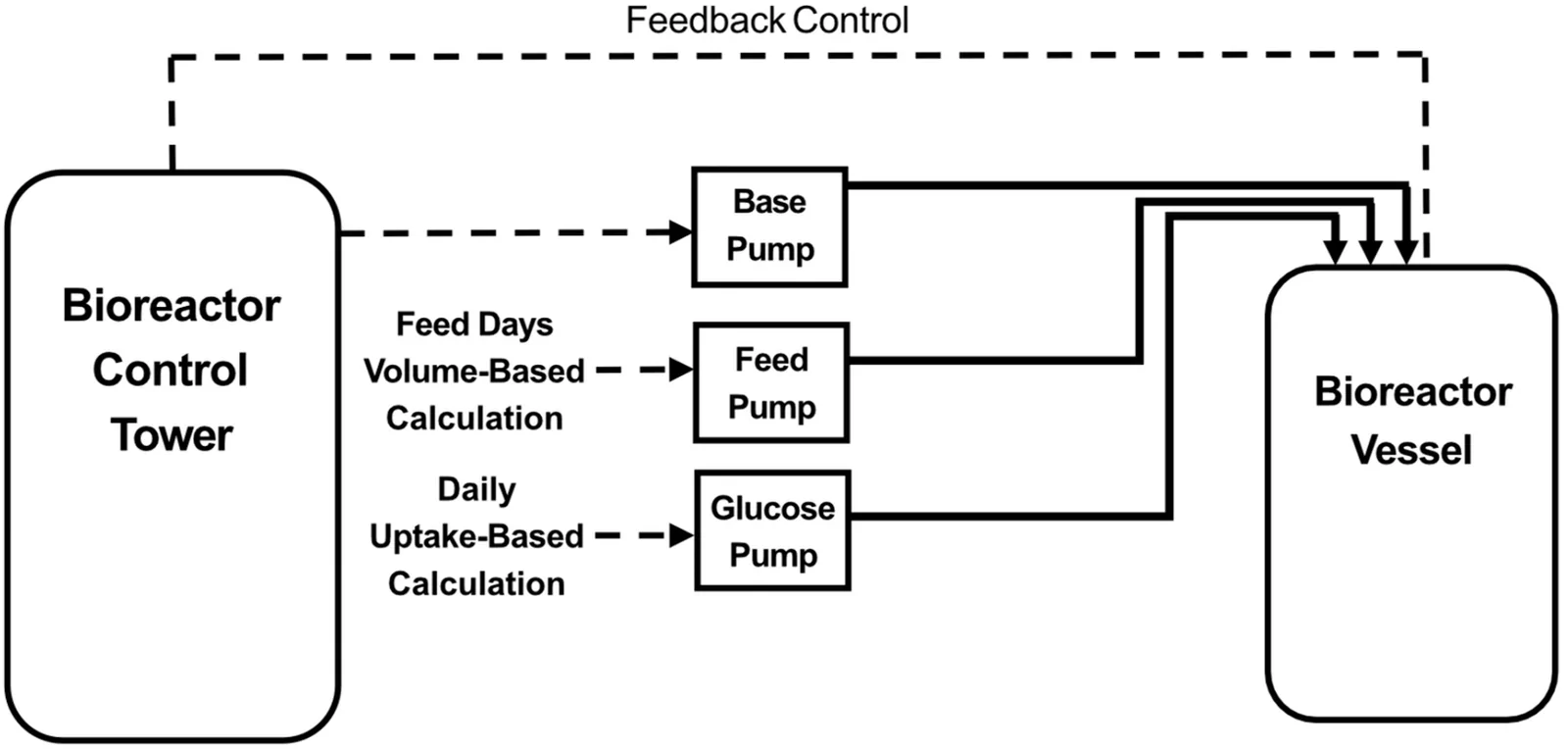
Fed batch diagram. Schematic for a fed-batch bioreactor set-up. Dotted lines depict feedback or operator-directed control. Solid lines with arrows depict the flow of fluid through the system.

In a study performed by Xu, et al., the seeding density was increased from∼6 × 10^5^ cells/mL to 1–2 × 10^7^ cells/mL. In comparison to the conventional fed-batch seed expansion with two batches before inoculation, the new process included an N-1 perfusion step to intensify the concentration of the cell culture (52, 53). The intensified process was successfully scaled up to a 500 L bioreactor for three mAbs and resulted in titers 10-fold higher than the tested conventional fed-batch process (57). The ability to obtain 10-fold higher titer results in the same amount of seed train steps will reduce COGs for each manufacturing run.

Perfusion is another technology that can be implemented to reduce COGs for a manufacturing process (56, 114). Perfusion is a continuous feed strategy that employs the constant removal of waste media and replacement with fresh media and feeds as shown in Figure 5. Cells are recirculated during perfusion, which allows for a higher VCD to be reached and maintained (56, 58, 113). A perfusion process enables scalable, longer, more stable production runs than the traditional fed-batch method while maintaining a high product quality (58, 100). Production can be extended while maintaining a high cell density, meaning more protein production per run, which will reduce overall manufacturing COGs (59, 60).

**Figure 5:**
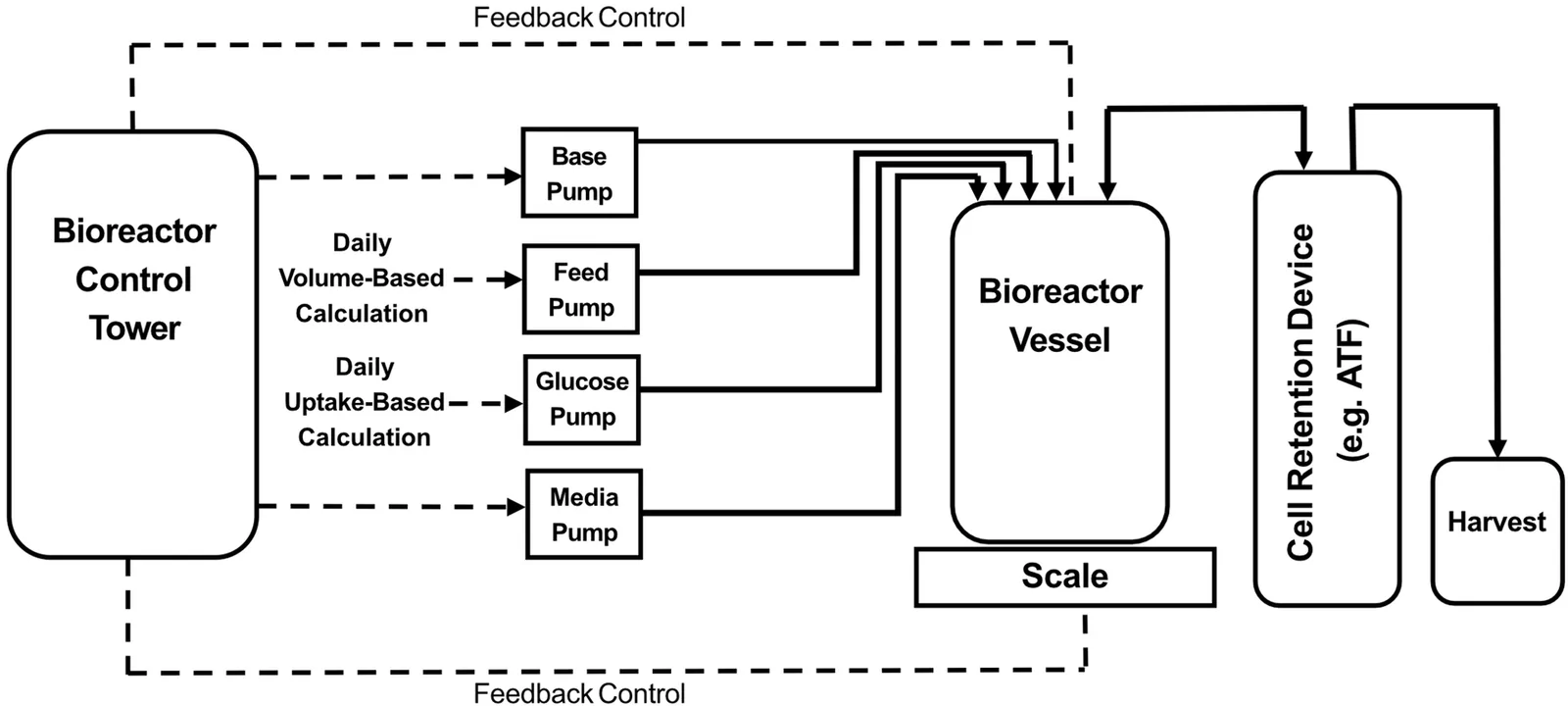
Perfusion diagram. Schematic for a perfusion bioreactor set-up. Dotted lines depict feedback or operator-directed control. Solid lines with arrows depict the flow of fluid through the system.

Utilizing perfusion, one study's yield per day increased to 1–3 grams/Liter/day from 0.3 g/L/day as seen with traditional fed-batch methods (60). This is a 3–10 fold increase in protein produced per day. Perfusion increases the stability of the culture to allow for higher cell densities to be maintained which in turn generated higher yields (61). VCDs from a traditional fed-batch process reached 2–3 × 10^7^ cells/mL, while perfusion reached 80–120 × 10^6^ cells/mL (60). In the Dorceus, et al. study, a 2.5-fold increase in product output was obtained using a perfusion method in comparison to the traditional fed-batch (59). This increase in production is reported in the literature with daily yields ranging from >1 g/L/d (62–64) to 5 g/L/d (65). While increasing the amount of necessary raw materials, this increase in production still reduces COGs required to manufacture the desired amount of protein and presents an opportunity to further exploit an already high-producing cell line.

A significant optimization opportunity for downstream purification exists in making yield improvements by reducing aggregates via optimized pH elution profiles in Protein A Purification. For Protein A affinity, elution is done in highly acidic conditions. For mAbs this can be a source of fragmentation and aggregation early in the process, which will cause a drop in yield (66). Performing design of experiments (DOE) studies to find an optimal pH that allows for the best yields possible, while also maintaining a pH that does not cause any quality issues to the product, could improve the quality of the mAb as it progresses through the downstream process and improve overall yield. Adding salts such as sodium chloride to reduce the need for harsher acidity in eluting, which can further improve yields (67), could help to serve this goal.

Historically, Protein A purification has accounted for roughly 25% of the total cost of mAb purification (68). Optimization of resin binding via residence time studies in Protein A columns is another route to optimization that can drastically reduce cost. Residence time is the measure of mAb contact time with resin. It can be honed to maximize mAb binding on the resin, reducing loss into the Flow Through (FT). This optimal contact can be determined by performing time trials with fixed column volumes and varying bed heights. Optimizing this variable is an essential precursor to one of the most promising cost saving technologies that could be utilized to streamline downstream production, Multi-Column Chromatography (MCC). MCC, depicted in Figure 6, is possible utilizing devices capable of shuffling interconnected columns with decreased bed volumes and allows for an increase in loading, a decrease in resin volume needed as the column is cycled repeatedly, and a reduction in the necessary time to perform the purification steps (50, 69). These devices enable a reduction in binding inefficiencies that often arise in single column processes. Instead of traditional loss to the FT due to either pumping too quickly or having an insufficient column height, any potential overloaded material is taken up by the next column in the sequence as opposed to that material being lost in traditional FT waste.

**Figure 6:**
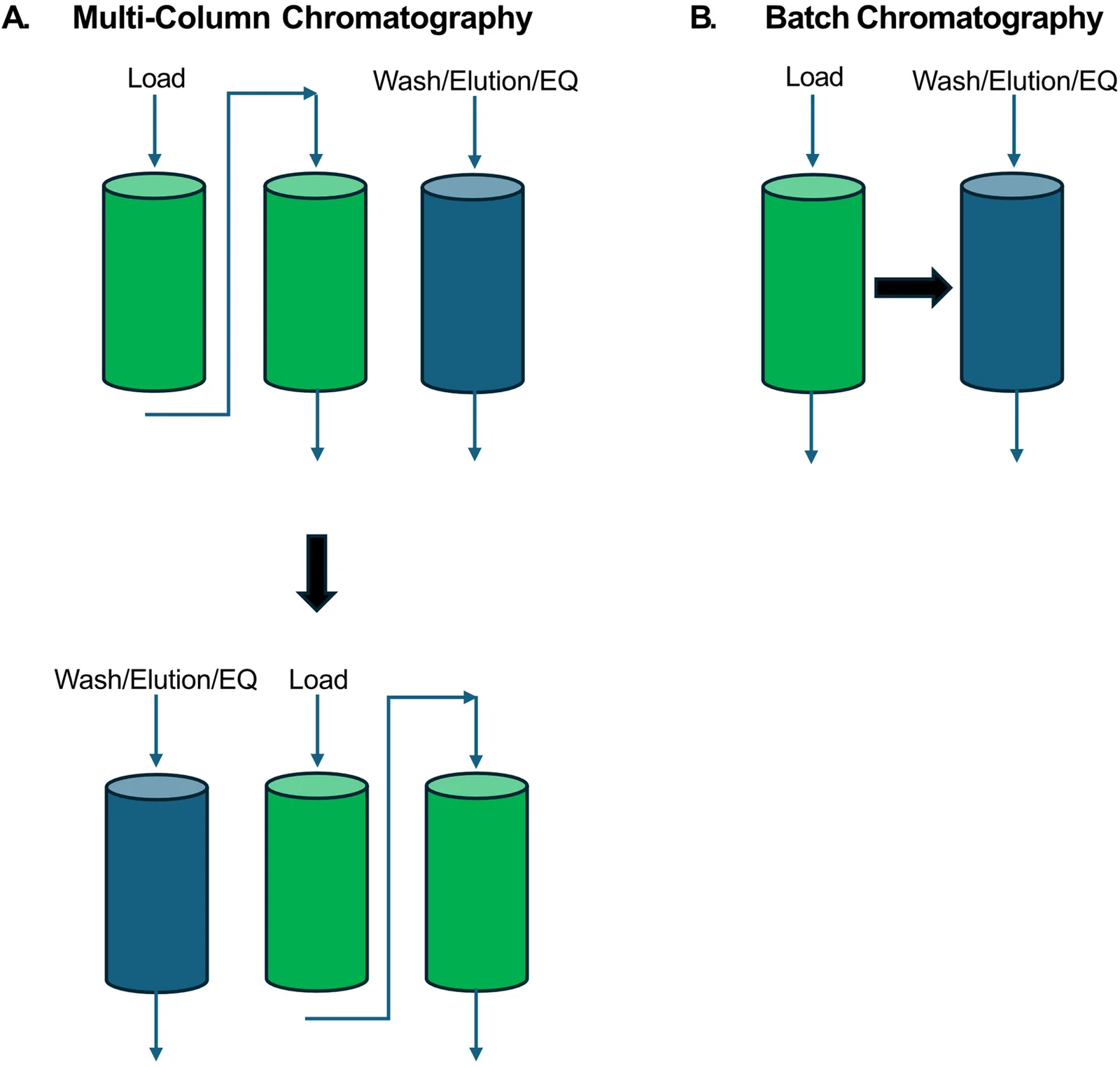
MCC and batch chromatography. **(A)** Multi-Column Chromatography (MCC) is a continuous purification process by which columns connected in series capture product breakthrough of the previous column. A more efficient flow path allows for lower buffer consumption, and higher binding capacity of resins such as Protein A affinity. In comparison, **(B)** Batch Chromatography is a single column process, which sees more product loss to the flowthrough, and lower Dynamic Binding Capacity (DBC) due to its limitations such as residence time and column length.

Additionally, the MCC setup saturates the resin in the columns more thoroughly with product, enabling better binding efficiency that is not possible in a single-column setup without risking material loss (70, 112). When utilizing Protein A resin specifically, studies have demonstrated a 4.6–5.1-fold increase in production efficiency than traditional batch chromatography and >72% reduction in total COGs per batch (70). When optimized these techniques should result in COGs savings aiding in bringing down the price of mAbs, potentially to levels needed for wider deployment in LMICs.

### Continuous manufacturing methodology

The approach proposed include the following modeling parameters:

1. High-productivity cell line. The model uses an ATUM miCHO cell line capable of achieving 6 grams/liter productivity, prior to any process development and bioreactor optimization.
2. Large scale continuous upstream, running ≥ 30,000 L of perfused cell culture and projected volumetric productivities of either 1.0, 3.0, or 5.0 grams/liter/day, also referred to as “low”, “moderate” and “optimistic” scenarios.
3. A semi-continuous, or continuous downstream processes, with recovery yields of ≥ 80%
4. A facility designed around the use of single use bioreactors at 2,000 L scale.
5. Facility and operations capable of successfully performing eight (8) successful manufacturing runs per year, including all equipment required to perform a continuous manufacturing process, and depreciation calculated based on an 8-year operating life.
6. Bulk purchasing and long-term supplier vendor contracts to reduce materials and consumables costs.
7. Commercial Scale Manufacturing in S. Africa. A new GMP facility and operation would be built around the use of continuous manufacturing of mAbs in single use production systems, with a skilled labor force projected at 98 scientific, managerial, technical, operational, and support professionals. Operating such a facility would be an organization domiciled in a relevant LMIC, for example an organization such as the South African Council on Scientific and Industrial Research (CSIR) would perform commercial scale mAbs manufacturing in South Africa.

The continuous manufacturing process proposed here details two scales of cell culture production runs (60,000 L and 30,000 L) for all three potential daily productivity rates, each broken out with a downstream yield at an 85% recovery and at an 80% recovery.

#### Modeling upstream process

To achieve a large scale continuous upstream process, the model utilizes a 2,000 L stirred tank bioreactor (STR). Two scale scenarios are represented in the Supplemental Materials, in which the vessel is either inoculated at a 1,000 L or 2,000 L initial working volume (IWV) utilizing material from a seed train encompassing shake flasks, a 50 L bioreactor, and a 200 L bioreactor for cell culture scale up. Utilizing an Alternating Tangential Flow Filtration (ATF) device to perform n-1 intensification on the 200 L vessel's material, the final 2,000 L vessel is inoculated, thus increasing the seed VCD and speeding up the timeframe necessary to reach a target VCD. After inoculation, the STR is allowed one day of growth to reach a target production VCD level before a temperature shift is initiated, and agitation is increased accordingly. An ATF system is connected to the STR to facilitate daily perfusion of 1.5 vessel volumes per day (VVD), continuously separating out healthy cells, removing spent media with harvest material and returning the cells to the reactor with replacement fresh media dosed with 7a and 7b supplements. All material harvested via this ATF process would be stored in a 3,000 L fluid storage tank, to proceed in subsequent downstream purification steps. This is repeated over the course of the remaining 20 days of the production run, equating to 30,000 L of total media perfused when the reactor is seeded at 1,000 L IWV and 60,000 L of total media perfused for a reactor seeded at 2,000 L IWV.

#### Modeling downstream process

Running concurrently with the above detailed upstream process, downstream purification would be performed on freshly perfused harvest material daily. For all purification steps, an FPLC system designed to facilitate Multi Column Chromatography would be employed to reduce the amount of necessary resin and allow for maximal usage of its binding capacity (105). The model budgets for three MCC FPLC systems, one for each purification step, enabling each step in the process to be performed daily, and removing the typical extended production run time seen in traditional fed-batch operations. All needed buffers will be manufactured in-house utilizing raw materials as indicated in the Supplemental Materials. Buffers will be formulated in the appropriately sized stainless-steel tanks and then sterile filtered through 0.2 μm filters into 3,000 L fluid storage tanks containing sterile bags. As several buffers require volumes in excess of 3,000 L, each buffer will be manufactured once and sterile filtered 3,000 L at a time into sterile storage tanks as necessary. Material stored in the 3,000 L fluid storage tank after perfusion from the STR is loaded onto one of the indicated FPLCs, prepared for Protein A purification. Table 3 indicates how this FPLC would be set up, with six columns packed with Protein A resin, adjusted according to production, and run size. The columns would be equilibrated with 5 CVs of 1× PBS pH 7.4 buffer. From there, the harvest material would be loaded on the first column with the remaining columns linked via the FPLC in sequence, until material was overloaded and began to run off on to the next column. This triggers an automated shift within the system of the first column to the washing stage while each subsequent column follows this same on-going loading process. After load completion, each column is washed with 5 CV's of 1× PBS pH 7.4. Following the wash step, 3 CV's of 2.41 mM Sodium Acetate pH 3.5 is applied as an elution buffer, and the eluate is collected in a 3,000 fluid storage tank. After each column is eluted, it will be completely stripped with 5 CVs of 0.1 M NaOH before being re-equilibrated to begin the process over again. The selected Protein A Resin has a dynamic binding capacity indicated by the supplier of 50 g/L, and testing done with ServareGMP's ZAC-3 mAb confirms this. The supplier also claims a 300-cycle lifespan of the Protein A resin while maintaining a dynamic binding capacity above 90% (71). Based on a 50 g/L binding capacity, and 300 cycle lifespan, the model projects cycling the entire system of 6 columns 15 times per day over the course of the 20 days that material is harvested from the STR. These conditions lead to a total of just over 294 necessary cycles per column per run, meaning the columns will not require repacking over the course of one full production run.

**Table 3:** Protein A column packing strategy for continuous manufacturing.

Production g/L/d	Total columns	60,000 L scale column volumes (L)	30,000 L scale column volumes (L)
5	6	3.4	1.7
3		2	1
1		0.67	0.335

After the entire days material has been successfully purified on Protein A resin, viral inactivation is performed by initiating a pH adjustment using 6.0 M HCl until a desired pH of 3.0–3.1 is achieved. A 30–45 min hold is then initiated to ensure complete viral inactivation. The viral inactivated material is then neutralized to a pH range of 6.95 to 7.05 using 1 M Tris-HCl pH 9.1.

Prior to beginning the next AEX step, the harvest material is diafiltered utilizing TFF in order to buffer exchange into AEX equilibration buffer, utilizing 6 diavolumes 20 mM Tris-HCl pH, 8.2.

The tank containing neutralized material is then connected to a second MCC FPLC instrument, set up for an AEX polishing step. Table 4 indicates how this FPLC would be setup, with columns packed with AEX resin adjusted according to production and run size. The AEX run is operated as a flowthrough step. Each column would be equilibrated utilizing 5 CVs 20 mM Tris-HCl pH, 8.2. After loading, 5 CVs of 20 mM Tris-HCl, 500 mM NaCl, pH 8.2 is utilized as elution/strip buffer. The selected AEX Resin has a dynamic binding capacity indicated by the supplier of 100 g/L. The supplier also claims a 50-cycle lifespan of the AEX resin. Based on a 100 g/L binding capacity, and 50 cycle lifespan, the model projects cycling the entire system of 6 columns 5 times per day over the course of the 20 days that material is harvested from the STR. These conditions lead to a total of 100 necessary cycles per column per run, meaning the columns will need to be repacked once after the 10th day of AEX purification. All of these calculations were performed without assuming any loss from the preceding steps, as this is not possible, nor what the model projects, this serves to ensure that these numbers will ultimately prove to be an overestimate of what is necessary. All flowthrough from this process, excluding the elution and strip steps, is collected in another 3,000 L fluid storage tank.

**Table 4:** AEX column packing strategy for continuous manufacturing.

Production g/L/d	Total columns	60,000 L scale column volumes (L)	30,000 L scale column volumes (L)
5	6	5	2.5
3		3	1.5
1		1	0.5

Prior to beginning the next CEX step, the harvest material is diafiltered utilizing TFF in order to buffer exchange into CEX equilibration buffer, utilizing 6 diavolumes 20 mM Sodium Acetate, pH 5.4.

A final MCC FPLC is attached to the above tank which is set up to perform a bind and elute CEX step. Table 5 demonstrates how this FPLC would be setup with columns packed with CEX resin adjusted according to production and run size. Following the column shuffling procedure, each column is equilibrated with 5 CVs of 20 mM Sodium Acetate, pH 5.4. Material is loaded onto the column and then washed using 5CVs of the equilibration buffer. A gradient elution is subsequently employed utilizing 20 mM Sodium Acetate, pH 5.4 as the “A Buffer” and 20 mM Sodium Acetate, 500 mM NaCl, pH 5.4 as the “B Buffer.” 3 CVs total should be passed over the column to complete this elution step. The selected CEX Resin has a Dynamic Binding Capacity (DBC) indicated by the supplier of 100 g/L. The supplier also claims a 50-cycle lifespan of the CEX resin. Based on a 100 g/L binding capacity, and 50 cycle lifespan, the model projects cycling the entire system of 6 columns 5 times per day over the course of the 20 days that material is harvested from the STR. These conditions lead to a total of 100 necessary cycles per column per run, meaning the columns will need to be repacked once after the 10th day of CEX purification. As with the AEX calculations, these were performed without assuming any loss from the preceding steps, as this is not possible, nor what the model projects, this serves to ensure that these numbers will be an overestimate of what is necessary. All eluted materials from this step are stored in a 3,000 L fluid storage tank.

**Table 5:** CEX column packing strategy for continuous manufacturing.

Production g/L/d	Total columns	60,000 L scale column volumes (L)	30,000 L scale column volumes (L)
5	6	5	2.5
3		3	1.5
1		1	0.5

This purified material is then passed through a Viral Filtration step using a filter train assembly incorporating a depth filter in series with a viral filter. After flushing and equilibrating the assembly, the CEX elution pool held in the storage tank is pumped through the filter train until all material has been processed.

To complete the downstream purification process, TFF is performed, ultrafiltering and diafiltering the material to concentrate the product to desired levels, and buffer exchange the material into the final fill or storage buffer. For cost projection purposes, 2.5 mM L-histidine, 5% sorbitol, 0.02% polysorbate 80, pH 6 was utilizes as an exemplar final formulation buffer in the Supplemental Materials.

### Continuous manufacturing discussion

The continuous manufacturing process outlined here, paired with the detailed COGs description presented in Supplemental Materials attempts to present a viable path to affordable mAb manufacturing. Pairing the high-producing cell lines with a 21-day perfusion process at large manufacturing scale, the model aims to outline a means of achieving the optimal possible yield, while taking advantage of discounted consumable pricing possible at those large volume orders. Due to the fluctuation in observed perfusion titers seen in the literature, this model also presents the three possible scenarios described above (optimistic performance, moderate performance, and low performance) to compare the potential impact varying yields could have on final cost of drug substance.

Continuous manufacturing provides the opportunity to utilize already needed consumables, such as resins, in a more economical manner. Examples include reducing the loss of high value mAbs, such as ZAC-3, to processing inefficiencies seen in factors such as DBC, resulting in mAb loss to the flow through. As well as reducing COGs associated with Protein A resin, re-cycling columns and shuffling them to theoretically extend bed height, further reductions in COGs are possible.

Single-use manufacturing caused a significant impact on bioprocessing, allowing manufacturers to reduce risks and improve workflows. Incorporating continuous manufacturing opens possibilities to reduce inefficiencies by developing a processing format that improves yield, while keeping the material footprint small compared to standard fed-batch processes of equivalent size.

### Continuous manufacturing conclusion

Historically, mAb manufacturing has presented challenges that resulted in prices that made cost-acceptable treatment in LMICs very difficult. Recent advances in CHO cell line engineering have produced significant increases in yield. Utilizing high performance cell lines and a large-scale, LMIC-based, continuous manufacturing approach decreases COGs compared with standard mAb manufacturing processes. As detailed in the scenario based economic model below, the proposed continuous manufacturing approach may aid in producing cost-effective mAbs, which would then enable treatment of a myriad of diseases impacting children and adults in LMICs.

## Section three: scenario based economic affordability model

### Introduction

To assist in developing attainable deployment of affordable mAbs for LMICs, we have created an economic model that projects costs to manufacture commercial ZAC-3 mAbs in very large quantities at an LMIC-sited GMP manufacturing operation. This model projects the cost of GMP manufacturing of bulk drug substance, or its COGs. As noted above, this model calculates cost to manufacture mAbs under optimistic, moderate, and low performance scenarios. These three scenarios all utilize the continuous manufacturing system as described above. Continuous manufacturing includes both upstream and downstream activities required to generate purified drug substance suitable for release for further manufacturing into drug product.

COGs are calculated for each of the three performance scenarios: the optimistic scenario projects a 5 grams/liter/day upstream productivity result, the moderate scenario at 3 grams/liter/day, and the low scenario at 1 grams/liter/day. These three scenarios utilize a perfusion production process described above and performed at run sizes of 30,000 L (30 k) and 60,000 L (60 k). Manufacturing runs include all upstream and downstream activities through the release of purified drug substance. 30 k and 60 k are the upstream scale manufacturing scales, but these scales are inclusive of all necessary downstream and drug substance release activities. The COGs breakdown for all scenarios and scales is provided below in Results.

A 60 k perfusion run scale is used to determine facility size, equipment needed, materials and components required, and personnel needed to perform these projected runs. A fourth scenario is provided which utilizes a high-performance fed-batch process at 2,000 L scale to provide a comparison between high performance fed batch and continuous manufacturing processes.

This model does not project the COGs to further manufacture mAbs into drug product or the cost to deploy mAbs for dosing of patients. This model is intended to calculate the COGs to manufacture drug substance and is not intended to calculate the price that would be achievable for drug dosing. Dose cost calculation requires determination of dose size, dosing method (i.e., intravenous infusion, intermuscular injection, or subcutaneous injection), and the costs to deliver a drug to the end point patient, which in turn requires determination of numerous factors such as stability of the drug, storage type (e.g., frozen, refrigerated, ambient), dosing locations (e.g., large city centralized hospital, or rural health clinic) and robustness of the delivery system, among many other factors. While an area ripe for further study, the COGs for drug product manufacturing and patient dosing cost needed to calculate the price of mAbs as drugs in LMICs are beyond the scope of this model.

### Scenario based economic model methodology

Our economic projection model is based on actual costs from vendors for equipment and materials needed to perform manufacturing; labor costs retrieved from published surveys for South African technical, support, and administration roles; and relevant facility operations indirect costs (e.g., indirect labor, facility leasing, utilities, insurance, maintenance, equipment depreciation). Actual costs are detailed in the Supplemental Materials. Total summary tables are provided below and in further detail in the Supplemental Materials. These costs are broken into three per run categories: materials and components, direct labor, and indirect costs, which are then combined for a total cost per gram to manufacture mAb drug substance.

#### Equipment, materials, and components

Costs for all equipment, materials, and components required to perform all aspects of the manufacturing runs at the various scales and performance levels detailed in this manuscript are compiled from publicly available vendor sources and special product purchase cost quotes provided by vendors from whom the materials and components would be purchased. Special order quotes were confidentially provided and therefore are semi-blinded to ensure compliance with applicable confidentiality terms. Where the term “Budgetary Quote” is used, vendors provided price per item and unit in another format. These items are detailed in Supplemental Materials, Vendor Price Sources Tab. Costs were obtained for performance of 60 k and 30 k scale. For actual performance of commercial scale campaigns, materials and components would be purchased on an annual basis with further reductions from those used in this model. However, as a cautious approach, the model uses costs on a per run basis. Regarding perfusion media, a $10 per liter at the 60 k order scale was provided by an international supplier as vendor in a confidential quote, and this cost per liter was also utilized in a 2021 study (19). As a contingency, this model uses an increased media cost to $13 per liter. All buffers needed to perform the proposed run scales are included in the model. Materials and components have a higher per unit cost for 30 k runs compared to 60 k runs and are included in each scale's COGs as such. Equipment cost is slightly lower for 30 k runs than for 60 k runs due to a slightly smaller equipment needed to perform manufacturing at that scale.

Labor costs. Labor personnel required to perform all aspects of commercial manufacturing runs are detailed Supplemental Materials. A full GMP mAbs manufacturing operation at 30 k and 60 k scale is determined to require 98 FTEs.

#### Direct and indirect labor

Plant management, scientific, technical, quality assurance and quality control, materials management, validations team, facilities and operations staff total 61 FTEs.

Indirect labor requires 37 administrative, accounting, security, IT, shipping and receiving, and maintenance total 37 FTEs for the 30 k and 60 k scale operations.

#### Total labor

All direct and indirect labor costs are totaled and shown in the Supplemental Material. Direct labor costs are provided separately from indirect labor in the full scope summary tables provided below and in the Supplemental Materials. Remuneration for the necessary roles is obtained from a survey of S. African survey sources, with a survey of US scientific staff compensation utilized as comparison (72–76). Necessary labor for 30 k scale manufacturing runs and 60 k manufacturing runs is the same and included in each run scale's COGs as such.

#### Indirect costs for operations

In addition to indirect labor, indirect costs to operate a commercial biologics manufacturing facility include Property Tax, Utilities, Maintenance, Insurance, Equipment, and Annual Facilities Lease.

#### Facility

This model projects a 6,000 square meter facility to perform drug substance manufacturing at the 30 k and 60 k scale. A market review of potentially suitable facilities in South Africa shows a lease range of between 85 rand and 110 rand per square meters per month (77, 78). This model uses 106 rand per square meter per month, which is the equivalent of $466,560 US per year (see Supplemental Material). Facility depreciation is not included in the COGs as facility ownership is not contemplated in this model. A facility size of 2,160 square meters is used for the fed-batch comparator cost model.

#### Calculations

Costs for materials, components, equipment, required for each run size are totaled and included in the COGs table calculation for both the 60 k run size and the 30 k run sizes under each scenario (optimistic, moderate and low). Costs for labor are totaled annually and then divided into per run costs per year, with this model assuming 8 runs per year at each of 60 k or 30 k scale. For this model, the maximum feasible facility usage is 10 manufacturing runs per year, thus allowing for two unsuccessful runs per year and 8 successful ones. Costs for all indirect categories are calculated as annual cost and then divided into a per run cost per year, again assuming 8 runs per year at each of 60 k and 30 k scales.

### Scenario based economic model results

The model calculated costs per gram of released drug substance under all four scenarios using the above methodology. The highest performing manufacturing process, the optimistic scenario (5 g/L/d), results in a projected $10.86 per gram cost at 60 k scale, 85% recovery, as shown below in Figure 7. The moderate scenario (3 g/L/d) results in in a projected $17.15 per gram cost at 60 k scale, 85% recovery, as shown below in Figure 8. The low performance scenario (1 g/L/d) results in in a projected $48.34 per gram cost at 60 k scale, 85% recovery, as shown below in Figure 9. Comparatively, a high-performance fed-batch manufacturing process, as described above and demonstrated in Figure 10, results in a per gram cost of $82.64 at 85% recovery.

**Figure 7:**
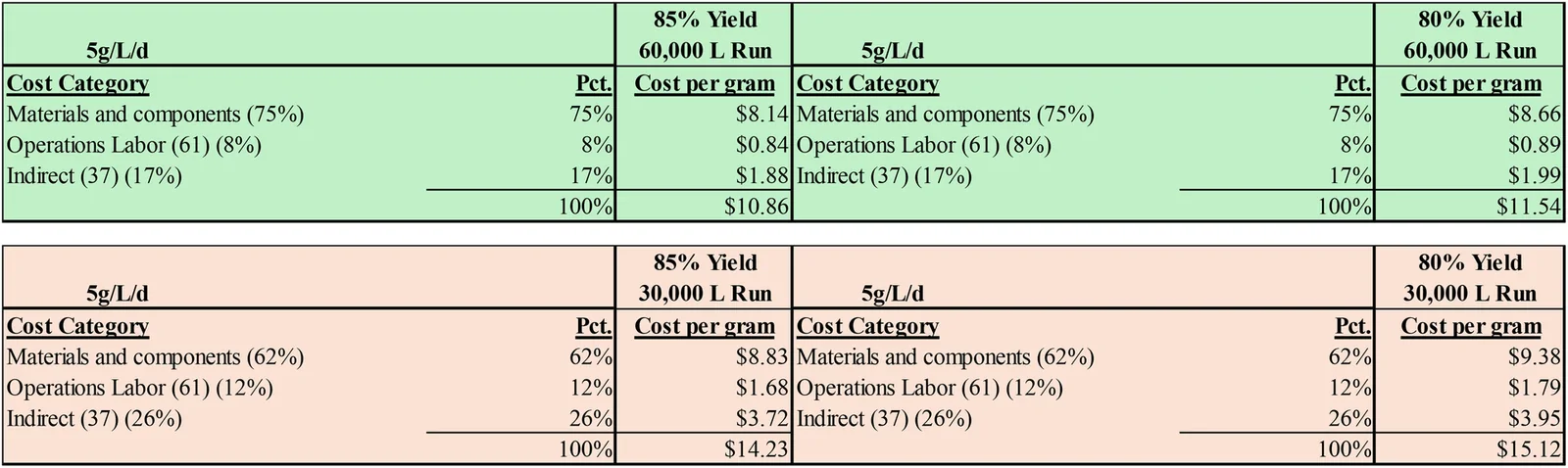
Optimistic performance scenario. 5 g per liter per day. Costs for elements needed to calculate per gram cost to manufacture mAb DS at 85% and 80% downstream recovery of clarified harvest, 60 k and 30 k production scale, at 5 g/L/d productivity.

**Figure 8:**
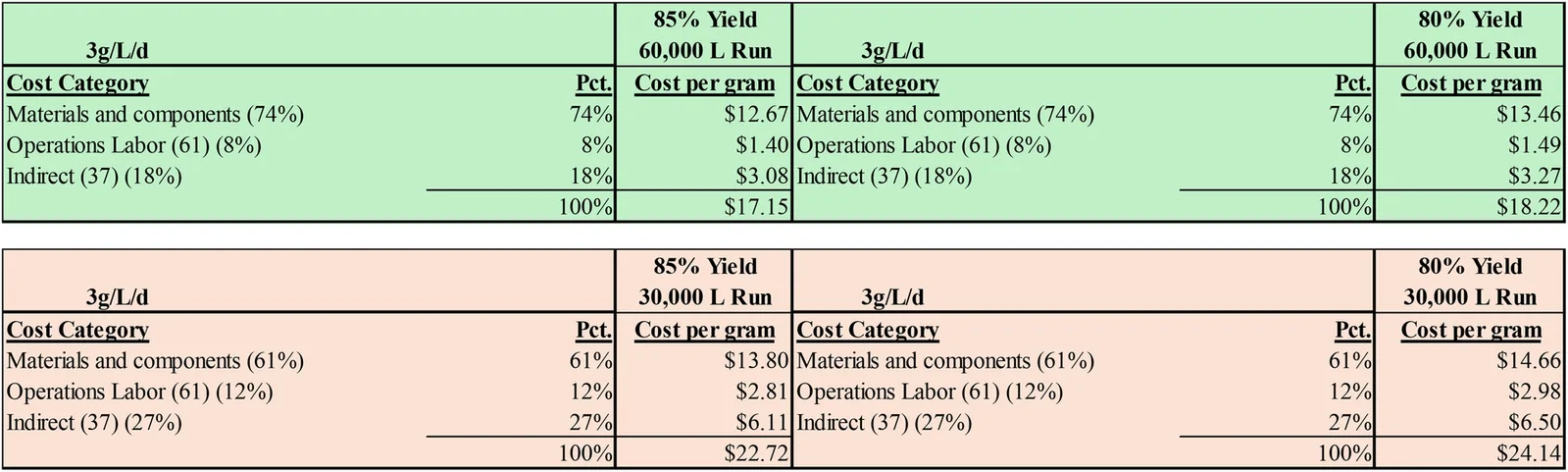
Moderate performance scenario. 3 g per liter per day. Costs for elements needed to calculate per gram cost to manufacture mAb DS at 85% and 80% downstream recovery of clarified harvest, 60 k and 30 k production scale, at 3 g/L/d productivity.

**Figure 9:**
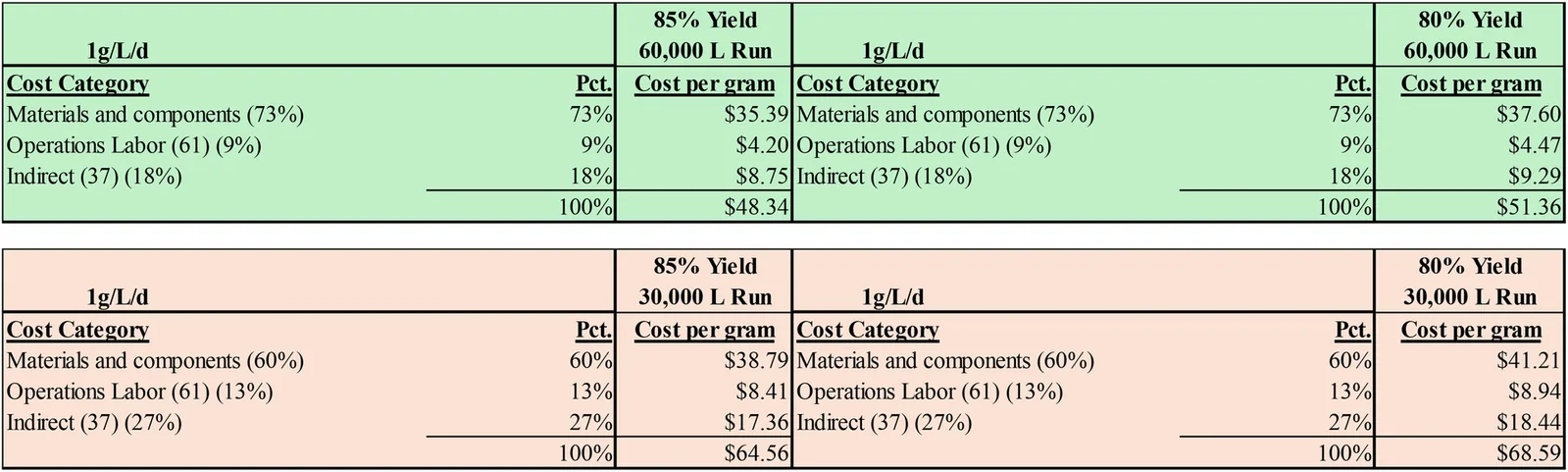
Low performance scenario. 1 g per liter per day. Costs for elements needed to calculate per gram cost to manufacture mAb DS at 85% and 80% downstream recovery of clarified harvest, 60 k and 30 k production scale, at 1 g/L/d productivity.

**Figure 10:**
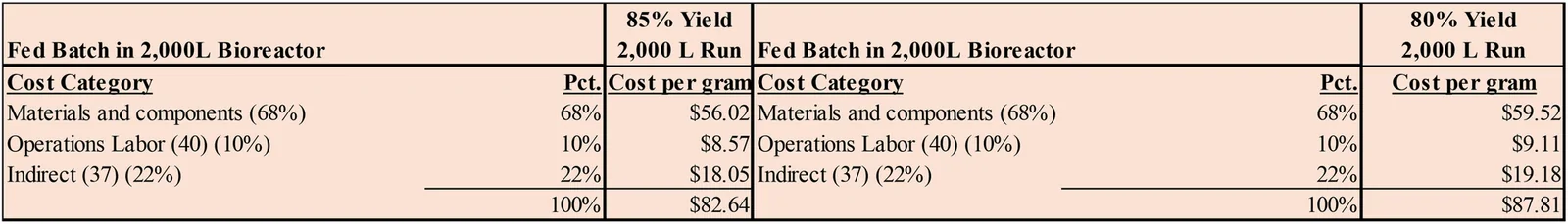
Comparator process fed-batch, 2,000 L production scale. Costs for elements needed to calculate per gram cost to manufacture mAb DS at 85% and 80% downstream recovery of clarified harvest, at 2,000 L fed-batch process, at 10 g/L.

The optimistic scenario process would generate 2.04 metric tons annually of purified released drug substance at 60 k scale, with an 85% recovery, at 8 runs per year. At 30 k scale, total production would be 1.02 metric tons. At 80% recovery, these run scales would produce 1.92 metric tons at 60 k scale and 0.96 metric tons at 30 k scale.

The moderate scenario process would generate annually 1.22 metric tons of purified released drug substance at 60 k scale, with an 85% recovery, at 8 runs per year. At 30 k scale, total production would be 0.612 metric tons. At 80% recovery, these run scales would produce 1.15 metric tons at 60 k scale and 0.576 metric tons at 30 k scale.

The low scenario process would generate annually 0.408 metric tons of purified released drug substance at 60 k scale, with an 85% recovery, at 8 runs per year. At 30 k scale, total production would be 0.204 metric tons. At 80% recovery, these run scales would produce 0.384 metric tons at 60 k scale and .192 metric tons at 30 k scale.

The Fed-batch 2,000 L comparator process would generate annually 0.136 metric tons of purified released drug substance at an 85% recovery, at 8 runs per year. At 80% recovery, a 2,000 L run would produce 0.128 annually.

### Scenario based economic model discussion

As shown above, under an 85% recovery after downstream processing, a continuous manufacturing process achieving a moderate performance level, could reduce the cost per gram to manufacture final drug substance compared to a high performance fed batch process nearly 5-fold in this model ($17.15 per gram compared to $82.64 per gram). A high-performance continuous manufacturing process could achieve a per gram cost of $10.86 per gram, a 7.6-fold reduction from the fed batch process. The reasons are: reductions in materials costs per unit under large volume ordering (e.g., 60 k liters perfusion media at $13 a liter); Protein A cost reduction using a continuous manufacturing initial capture instead of single column processing; staff and operation efficiencies where 98 staff projected to perform 60 k perfusion runs vs. 77 staff required to perform 2,000 L fed batch runs; equipment projected at $9.3 m for 60 k perfusion run vs. $6.8 m for 2,000 L fed batch runs; and a facility to perform 60 k (or 30 k) perfusion runs, while larger than a facility to perform 2,000 L fed batch runs, is projected at 6 k square meters vs. 2 k square meters for the 2,000 L capable facility, and not 15 to 30 times larger. All these factors result in lower cost per gram in both 30 k and 60 k perfusion runs under both the moderate and optimistic scenarios.

However, with the low performing scenario, the lower cost per gram is not nearly as great as those of the moderate and optimistic scenarios, with only a 1.8-fold decrease over the 2,000 L fed batch process. With either larger scale fed batch bioreactor scales (4,000 L–6,000 L scale) or a higher productivity (e.g., 14/g per liter) the difference could be partially or fully offset.

### Scenario based economic model conclusion

The model detailed in this paper shows that a high-performance continuous manufacturing process, and even a moderate performance process, could achieve the cost per gram of drug substance needed to achieve a potential deployment of mAbs in moderate and even low-income countries. While the cost of further manufacturing into drug product and especially deploying drug doses to certain hard to reach areas (e.g., rural clinics) are a meaningful part of the overall cost to provide patients in need medicines they deserve, cost calculations of those items are beyond the scope of this paper. A comprehensive assessment of those costs would add valuable knowledge in the effort to provide medicines to peoples in need. That being said, achieving very low cost per gram of mAb drug substance is the first step in mitigating that need.

## Manuscript conclusion

The purpose of this hybrid manuscript is to show that a novel mAb called ZAC-3 is effective against the bacterium *V. cholerae*; that a proposed continuous manufacturing process, building off the existing ZAC-3 fed-batch manufacturing process, has the potential for generating very large amounts of drug substance utilizing 30 k and 60 k scale perfusion runs in a 2,000 L bioreactor platform; and that an economic model based on that continuous manufacturing process provides a path to manufacture mAbs at a very low cost that may translate into the low dose prices needed for mAbs to be successfully deployed for patients in low and moderate income countries, particularly if such manufacturing occurs in LMICs such as South Africa. There are a number of questions to answer, such as whether a perfusion production process can achieve the performance results included in the model in the optimistic scenario, whether further manufacturing of drug substance into drug product and the costs associated with deployment to actual patients would result in an unaffordable price for a mAb such as ZAC-3. However, we believe that the information and data provided in this manuscript lends credence to the position that low price mAbs can be deployed in LMICs.

## Data Availability

The original contributions presented in the study are included in the article/Supplementary Material, further inquiries can be directed to the corresponding authors.

## References

[1] Food and Drug Administration (FDA). Interpreting sameness of monoclonal antibody products under the orphan drug regulations. Available online at: https://www.fda.gov/files/vaccines,%20blood%20&%20biologics/published/Interpreting-Sameness-of-Monoclonal-Antibody-Products-Under-the-Orphan-Drug-Regulations.pdf (Accessed January 14, 2026).

[2] Kothari M Wanjari A Acharya S Karwa V Chavhan R Kumar S. A comprehensive review of monoclonal antibodies in modern medicine: tracing the evolution of a revolutionary therapeutic approach. Cureus. (2024) 16(6):e61983. 10.7759/cureus.61983 38983999 PMC11231668

[3] Larossa C Mora J Cheung N-K. Global impact of monoclonal antibodies (mAbs) in children: a focus on anti-GD2. Cancers (Basel). (2023) 15(14):3729. 10.3390/cancers15143729 37509390 PMC10378537

[4] Malik B Ghatol A. Understanding how Monoclonal Antibodies Work. Treasure Island (FL): StatPearls Publishing (2023). Available online at: https://www.ncbi.nlm.nih.gov/books/NBK572118/ (Accessed March 26, 2026). 34283484

[5] Kelley B. The history and potential future of monoclonal antibody therapeutics development and manufacturing in four eras. mAbs. (2024) 16(1). 10.1080/19420862.2024.2373330. 38946434 PMC11218797

[6] Shepard HM Phillips GL Thanos CD Feldmann M. Developments in therapy with monoclonal antibodies and related proteins. Clin Med (Lond). (2017) 17(3):220–32. 10.7861/clinmedicine.17-3-220 28572223 PMC6297577

[7] Lu R-M Chiang H-L Yuan JP-Y Wang H-H Chen C-Y Panda SS. Technological advancements in antibody-based therapeutics for treatment of diseases. J Biomed Sci. (2025) 32(1):98. 10.1186/s12929-025-01190-2 41219972 PMC12606834

[8] Strohl WR. Structure and function of therapeutic antibodies approved by the US FDA in 2024. Antibody Therapeutics. (2025) 8(3):197–237. 10.1093/abt/tbaf014 40994925 PMC12454936

[9] Markets and Markets. Monoclonal Antibody (mAb) Therapeutics Market Worth $497.5 billion. 2026 March 14. Available online at: https://www.prnewswire.com/news-releases/monoclonal-antibody-mabs-therapeutics-market-worth-497-5-billion-marketsandmarkets-302090362.html (Accessed April 3, 2026).

[10] Whaley KJ Zeitlin L. Emerging antibody-based products for infectious diseases: planning for metric ton manufacturing. Hum Vaccin Immunother. (2022) 18(2). 10.1080/21645515.2021.1930847. 34259613 PMC9103258

[11] Precedence Research. Antibody Therapy Market to Surge to USD 951.24 Billion by 2034 at 13.08% CAGR Towards Healthcare. Ottawa: GlobeNewswire (2025). Available online at: https://www.globenewswire.com/news-release/2025/10/22/3171251/0/en/antibody-therapy-market-to-surge-to-usd-951-24-billion-by-2034-at-13-08-cagr-towards-healthcare.html (Accessed April 3, 2026).

[12] Xenopoulos A. A new, integrated, continuous purification process template for monoclonal antibodies: process modeling and cost of goods studies. J Biotechnol. (2015) 213:42–53. 10.1016/j.jbiotec.2015.04.020 25959171

[13] India Vaccination Centre. Beyfortus Immunization in Noida. Noida (IN): InvC (2026). Available online at: https://invc.co.in/rsv/beyfortus-immunization/noida (Accessed February 2, 2026).

[14] Pérez Casas C Tagarro A Bassat Q Rojo P. The need for RSV prophylaxis in LMICs. Pediatrics. (2024) 154(4):e2024067062. 10.1542/peds.2024-067062. Available online at: https://publications.aap.org/pediatrics/article/154/4/e2024067062/199330/The-Need-for-RSV-Prophylaxis-in-LMICs (Accessed February 2, 2026). 39252637

[15] Guiñazú G Dvorkin J Mahmud S Baral R Pecenka C Libster R. Evaluation of the potential impact and cost-effectiveness of respiratory syncytial virus (RSV) prevention strategies for infants in Argentina. Vaccine. (2024) 42(23):126234. 10.1016/j.vaccine.2024.126234 PMC11413482 39154512

[16] Do LAH Le NTN Mahmud S Mulholland K Pecenka C Clark A. Impact and cost-effectiveness of strategies to prevent respiratory syncytial virus (RSV) disease in Vietnam: a modelling study. Vaccine. (2023) 41(46):6782–90. 10.1016/j.vaccine.2023.09.003 37690873 PMC10626342

[17] American Academy of Pediatrics. RSV Immunization Products & Ordering Information. (Last updated on 2025 September 9). Available online at: https://www.aap.org/en/patient-care/respiratory-syncytial-virus-rsv-prevention/nirsevimab-beyfortus-product-ordering-information/?srsltid=AfmBOor8m0HXjf9p7pI9tzXmw4cIy2emSYRRo9u_VSs949cilZ2A41Vo (Accessed January 14, 2026).

[18] Malhotra S Cameron A-I Gotham D Burrone E Gardner PJ Loynachan C. Novel approaches to enable equitable access to monoclonal antibodies in low- and middle-income countries. PLOS Global Public Health. (2024) 4(7):e0003418. 10.1371/journal.pgph.0003418 38950021 PMC11216602

[19] Mahal H Branton H Farid SS. End-to-end continuous bioprocessing: impact on facility design, cost of goods, and cost of development for monoclonal antibodies. Biotechnol Bioeng. (2021) 118(9):3468–85. 10.1002/bit.27774 33792918

[20] Boutayeb A. Developing countries and neglected diseases: challenges and perspectives. Int J Equity Health. (2007) 6:20. 10.1186/1475-9276-6-20 18036265 PMC2206025

[21] Gieber L Muturi-Kioi V Malhotra S Sitlani A. Clinical and regulatory challenges and opportunities for monoclonal antibodies in low- and middle-income countries: lessons from COVID-19 and beyond. Pharmaceut Med. (2023) 37(3):203–14. 10.1007/s40290-023-00473-z 37115494 PMC10141866

[22] Health Tech Africa. Monoclonal antibodies in healthcare. Available online at: https://healthtechafrica.org/technology/monoclonal-antibodies-healthcare (Accessed April 2, 2026).

[23] Hernandez I Bott SW Patel AS Wolf CG Hospodar AR Sampathkumar S. Pricing of monoclonal antibody therapies: higher if used for cancer? Am J Manag Care. (2018) 24(2):109–12. (Accessed April 3, 2026). 29461857

[24] Birch JR Racher AJ. Antibody production. Adv Drug Deliv Rev. (2006) 58(5–6):671–85. 10.1016/j.addr.2005.12.006 16822577

[25] Chen C Garcia Z Chen D Liu H Trelstad P. Cost and supply considerations for antibody therapeutics. MAbs. (2025) 17(1):2451789. 10.1080/19420862.2025.2451789 39829035 PMC12952249

[26] Sagandira CR. Agile local manufacturing of active pharmaceutical ingredients in Africa could improve health security and economic growth. Commun Med (Lond). (2025) 5(1):358. 10.1038/s43856-025-01080-6 40830584 PMC12365005

[27] Yieke L. Momentum Builds for Local Drug Production. London: African Business (2026). Available online at: https://african.business/2026/04/trade-investment/momentum-builds-for-local-drug-production (Accessed April 9, 2026).

[28] Haque H Rahim FO Munshi ZR Sandhu M Ojelade T Khan E. Strategic pathways to pharmaceutical self-reliance in Africa: lessons, enablers, and opportunities. J Glob Health Econ Policy. (2026) 5:e2025049. 10.7189/001c.154008

[29] Harris JB LaRocque RC Qadri F Ryan ET Calderwood SB. Cholera. Lancet. (2012) 379(9835):2466–76. 10.1016/s0140-6736(12)60436-x 22748592 PMC3761070

[30] Montero DA Vidal RM Velasco J George S Lucero Y Gómez LA. *Vibrio cholerae*, classification, pathogenesis, immune response, and trends in vaccine development. Front Med (Lausanne). (2023) 10. 10.3389/fmed.2023.1155751. PMC10196187 37215733

[31] Iyer AS Harris JB. Correlates of protection for cholera. J Infect Dis. (2021) 224(Supplement_7):S732–7. 10.1093/infdis/jiab497 34668561 PMC8687088

[32] Charles RC Kelly M Tam JM Akter A Hossain M Islam K. Humans surviving cholera develop antibodies against *vibrio cholerae* O-specific polysaccharide that inhibit pathogen motility. mBio. (2020) 11(6). 10.1128/mbio.02847-20. 33203761 PMC7683404

[33] Holmgren J. An update on cholera immunity and current and future cholera vaccines. Trop Med Infect Dis. (2021) 6(2):64. 10.3390/tropicalmed6020064 33925118 PMC8167659

[34] Leitner DR Feichter S Schild-Prüfert K Rechberger GN Reidl J Schild S. Lipopolysaccharide modifications of a cholera vaccine candidate based on outer membrane vesicles reduce endotoxicity and reveal the major protective antigen. Infect Immun. (2013) 81(7):2379–93. 10.1128/iai.01382-12 23630951 PMC3697601

[35] Arencibia-Mohar A Ariosa-Alvarez A Madrazo-Alonso O González Abreu E Garcı´a-Imia L Sierra-González G. Synthesis of terminal disaccharide elements corresponding to the ogawa and inaba antigenic determinant from *Vibrio cholerae* O1. Carbohydr Res. (1998) 306(1–2):163–70. 10.1016/s0008-6215(97)10052-0 9691445

[36] Levinson KJ Baranova DE Mantis NJ. A monoclonal antibody that targets the conserved core/lipid a region of lipopolysaccharide affects motility and reduces intestinal colonization of both classical and El Tor *Vibrio cholerae* biotypes. Vaccine. (2016) 34(48):5833–6. 10.1016/j.vaccine.2016.10.023 27773473 PMC5193159

[37] Baranova DE Willsey GG Levinson KJ Smith C Wade J Mantis NJ. Transcriptional profiling of *vibrio cholerae* O1 following exposure to human anti- lipopolysaccharide monoclonal antibodies. Pathog Dis. (2020) 78(4). 10.1093/femspd/ftaa029. 32589220 PMC7371154

[38] Bahadir Z Narayan P Wolters R Permar SR Fouda G Hessell AJ. Monoclonal antibodies for pediatric viral disease prevention and treatment. Pediatrics. (2025) 155(5):e2024068690. 10.1542/peds.2024-068690 40174915 PMC13020762

[39] Majumdar S Desai R Hans A Dandekar P Jain R. From efficiency to yield: exploring recent advances in CHO cell line development for monoclonal antibodies. Mol Biotechnol. (2025) 67(2):369–92. 10.1007/s12033-024-01060-6 38363529

[40] Ranbhor R. Advancing monoclonal antibody manufacturing: process optimization, cost reduction strategies, and emerging technologies. Biologics. (2025) 19:177–87. 10.2147/BTT.S515078 40226587 PMC11994081

[41] Dabrowski P Rasmus M Jundzill A Drewa T Pokrywczynska M. A comparison of five methods to maximize RNA and DNA isolation yield from adipose tissue. PeerJ. (2024) 12:e17071. 10.7717/peerj.17071 38711623 PMC11073010

[42] Porterfield JZ Zlotnick A. A simple and general method for determining the protein and nucleic acid content of viruses by UV absorbance. Virology. (2010) 407(2):281–8. 10.1016/j.virol.2010.08.015 20850162 PMC2964155

[43] Jing S-Y Gou J-X Gao D Wang H-B Yao S-J Lin D-Q. Separation of monoclonal antibody charge variants using cation exchange chromatography: resins and separation conditions optimization. Sep Purif Technol. (2020) 235:116136. 10.1016/j.seppur.2019.116136

[44] Doering JE Adewunmi Y Deal CE Plante O Carfi A Mantis NJ. Maternal expression and breast milk transfer of an mRNA- encoded monoclonal antibody in a murine model of cholera. PLoS Negl Trop Dis. (2026) 20(2):e0013518. 10.1371/journal.pntd.0013518 41662412 PMC12919922

[45] Lindberg SK Willsey GG Mantis NJ. Flagellar-based motility accelerates IGA-mediated agglutination of salmonella typhimurium at high bacterial cell densities. Front Immunol. (2023) 14. 10.3389/fimmu.2023.1193855 37275888 PMC10232770

[46] Regeneron Pharmaceuticals, Inc. Regeneron announces new U.S. government agreement to purchase additional doses of REGEN-COV™ (casirivimab and imdevimab) antibody cocktail 2021 September 14. Available online at: https://investor.regeneron.com/news-releases/news-release-details/regeneron-announces-new-us-government-agreement-purchase (Accessed March 8, 2026).

[47] Farid SS Baron M Stamatis C Nie W Coffman J. Benchmarking biopharmaceutical process development and manufacturing cost contributions to R&D. mAbs. (2020) 12(1):1754999. 10.1080/19420862.2020.1754999 32449439 PMC7531566

[48] Farid SS Washbrook J Titchener-Hooker NJ. Decision-support tool for assessing biomanufacturing strategies under uncertainty: stainless steel versus disposable equipment for clinical trial material preparation. Biotechnol Prog. (2005) 21(2):486–97. 10.1021/bp049692b 15801789

[49] Klutz S Magnus J Lobedann M Schwan P Maiser B Niklas J. Developing the biofacility of the future based on continuous processing and single-use technology. J Biotechnol. (2015) 213:120–30. 10.1016/j.jbiotec.2015.06.388 26091773

[50] Rathore AS Metya S Nitika N. Do we really need end-to-end continuous processing for biomanufacturing of monoclonal antibodies? Biotechnol Bioeng. (2025) 123(3):792–800. 10.1002/bit.70147 41457432

[51] Padawer I Ling WLW Bai Y. Case study: an accelerated 8-day monoclonal antibody production process based on high seeding densities. Biotechnol Prog. (2013) 29:829–32. 10.1002/btpr.1719 23596148

[52] Stepper L Filser FA Fischer S Schaub J Gorr I Voges R. Pre-stage perfusion and ultra-high seeding cell density in CHO fed-batch culture: a case study for process intensification guided by systems biotechnology. Bioprocess Biosyst Eng. (2020) 43:1431–43. 10.1007/s00449-020-02337-1 32266469 PMC7320070

[53] Xu J Xu X Huang C Angelo J Oliveira CL Xu M. Biomanufacturing evolution from conventional to intensified processes for productivity improvement: a case study. mAbs. (2020) 12(1). 10.1080/19420862.2020.1770669. PMC7531520 32425110

[54] Khanal O Lenhoff AM. Developments and opportunities in continuous biopharmaceutical manufacturing. mAbs. (2021) 13(1). 10.1080/19420862.2021.1903664. 33843449 PMC8043180

[55] Cunningham M Mahadevan J Bausch M. Optimizing CHO cells for higher productivity. (August 2020). MilliporeSigma. Available online at: https://www.sigmaaldrich.com/US/en/technical-documents/technical-article/pharmaceutical-and-biopharmaceutical-manufacturing/monoclonal-antibody-manufacturing/higher-productivity-cho-cell (Accessed March 6, 2026).

[56] MilliporeSigma. Evaluation of novel cell retention technology and cell culture media for N-1 perfused seed train. (2010). Available online at: https://www.sigmaaldrich.com/deepweb/assets/sigmaaldrich/product/documents/257/539/pro-intensified-processing-an5927en-ms.pdf?srsltid=AfmBOooPrk_YJxysMTgaFg35Td9YZnyPm7hppnUJEJSIDJRE94l6F3as (Accessed February 9, 2026).

[57] Xu J Rehmann MS Xu M Zheng S Hill C He Q. Development of an intensified fed-batch production platform with doubled titers using N-1 perfusion seed for cell culture manufacturing. Bioresour Bioprocess. (2020) 7(17). 10.1186/s40643-020-00304-y.

[58] Liang K Luo H Li Q. Enhancing and stabilizing monoclonal antibody production by Chinese hamster ovary (CHO) cells with optimized perfusion culture strategies. Front Bioeng Biotechnol. (2023) 11:1112349. 10.3389/fbioe.2023.1112349 36741761 PMC9895834

[59] Dorceus M Willard SS Suttle A Han K Chen PJ Sha M. Comparing culture methods in monoclonal antibody production: batch, fed-batch, and perfusion. BioProcess Int. (2017). Available online at: https://www.bioprocessintl.com/process-development/comparing-culture-methods-in-monoclonal-antibody-production-batch-fed-batch-and-perfusion (Accessed February 17, 2026).

[60] Gibco cell culture for bioprocessing. Savings that speak volumes: the economic benefits of perfusion bioprocessing. Thermo Fisher Scientific. Available online at: https://documents.thermofisher.com/TFS-Assets/BPD/Reference-Materials/Economic-Benefits-of-Perfusion-white-paper.pdf (Accessed February 13, 2026).

[61] Yang WC Lu J Kwiatkowski C Yuan H Kshirsagar R Ryll T. Perfusion seed cultures improve biopharmaceutical fed-batch production capacity and product quality. Biotechnol Prog. (2014) 30:616–25. 10.1002/btpr.1884 24574326

[62] Ott V Ott J Eibl D Eibl R. Scaling fed-batch and perfusion antibody production processes in geometrically dissimilar stirred bioreactors. Processes. (2024) 12(4):806. 10.3390/pr12040806

[63] Gagnon M Nagre S Wang W Hiller GW. Shift to high-intensity, low-volume perfusion cell culture enabling a continuous, integrated bioprocess. Biotechnol Prog. (2018) 34(6):1472–81. 10.1002/btpr.2723 30298995

[64] Xu S Gavin J Jiang R Chen H. Bioreactor productivity and media cost comparison for different intensified cell culture processes. Biotechnol Prog. (2017) 33(4):867–78. 10.1002/btpr.2415 27977910

[65] Sun T Zhang Y Liang H Fang W Qian Z Tan KW. Establishment of a semi-continuous scale-down clone screening model for intensified perfusion culture. Biotechnol Lett. (2024) 46(6):1085–93. 10.1007/s10529-024-03512-0 39066960

[66] Li Z Hu L Li Y. The advantage of high pH eluting Protein A resins over their regular counterparts in aggregate and host cell protein clearance. Protein Expression Purif. (2026) 239:106859. 10.1016/j.pep.2025.106859 41260415

[67] Zhang Y Wang Y Li Y. A method for improving Protein A chromatography’s aggregate removal capability. Protein Expression Purif. (2019) 158:65–73. 10.1016/j.pep.2019.02.017 30831226

[68] Broly H Costioli MD Guillemot-Potelle C Mitchell-Logean C. Cost of goods modeling and quality by design for developing cost-effective processes. BioPharm Int. (2010) 23(6):26–35. Available online at: https://www.biopharminternational.com/view/cost-goods-modeling-and-quality-design-developing-cost-effective-processes (Accessed March 2, 2026).

[69] Shukla AA Wolfe LS Mostafa SS Norman C. Evolving trends in monoclonal antibody production processes. Biotechnol Transl Med. (2017) 2(1):58–69. 10.1002/btm2.10061 PMC5689530 29313024

[70] Kulkarni SS. Advances in fermentation-based production systems and downstream processing strategies. BioProcess J. (2015) 25. Available online at: https://bioprocessingjournal.com/afp/J25OA-Kulkarni.pdf (Accessed March 3, 2026).

[71] JSR Life Sciences. Protein A Affinity Resin Amsphere A3. Sunnyvale, CA: JSR Life Sciences (2024). Available online at: https://dl.novachem.com.au/files/AmsphereA3.pdf (Accessed May 12, 2026).

[72] Statistics South Africa. Quarterly Employment Statistics (QES), June 2024. Pretoria (ZA): Statistics South Africa (2024). Available online at: https://www.statssa.gov.za/publications/P0277/P0277June2024.pdf (Accessed March 1, 2026).

[73] U.S. Bureau of Labor Statistics. Current Employment Statistics (CES): Employment and Earnings Data, 2024. Washington (DC): U.S. Bureau of Labor Statistics (2024). Available online at: https://www.bls.gov/ces/data/employment-and-earnings/2024/ (Accessed March 1, 2026).

[74] Academic Positions. PhD, professor, and postdoc salaries in the United States in 2024. Academic Positions (2024). Available online at: https://academicpositions.com/career-advice/phd-professor-and-postdoc-salaries-in-the-united-states-in-2024 (Accessed March 2, 2026).

[75] National Treasury of South Africa. Medium Term Budget Policy Statement (MTBPS) Annexure: Compensation and Employment Data. Pretoria (ZA): National Treasury (2025). Available online at: https://www.treasury.gov.za/documents/mtbps/ (Accessed March 2, 2026).

[76] Department of Trade, Industry and Competition. Facts and Figures on Skills in Manufacturing. Pretoria (ZA): dtic (2017. Available online at: https://www.thedtic.gov.za/wp-content/uploads/publication-FACTS_FIGURES.pdf (Accessed March 2, 2026).

[77] Private Property. 19,162m^2^ Industrial space in Industria West. Available online at: https://www.privateproperty.co.za/commercial-sales/gauteng/johannesburg/johannesburg-central/industria-west/50-commando-road/T5443207 (Accessed May 10, 2026).

[78] Spire Property Solutions. 125 industrial properties to let | Spire Property Solutions. Available online at: https://www.spire.co.za/results/industrial/to-let/ (Accessed May 10, 2026).

[79] Schwartz NRM Flanagan MR Babigumira JB Steuten LM Roth JA. Cost-effectiveness analysis of adjuvant neratinib following trastuzumab in early-stage HER2-positive breast cancer. J Manag Care Spec Pharm. (2019) 25(10):1133–9. 10.18553/jmcp.2019.25.10.1133 31556818 PMC10398073

[80] GoodRx. Humira: how much is Humira without insurance? Available online at: https://www.goodrx.com/humira/how-much-is-humira-without-insurance (Accessed March 2, 2026).

[81] Merck & Co., Inc. KEYTRUDA® (pembrolizumab): financial support. Available online at: https://www.keytruda.com/financial-support/ (Accessed March 3, 2026).

[82] American Academy of Pediatrics. Nirsevimab (Beyfortus) product and ordering information. Available online at: https://www.aap.org/en/patient-care/respiratory-syncytial-virus-rsv-prevention/nirsevimab-beyfortus-product-ordering-information/ (Accessed February 17, 2026).

[83] Cancer Association of South Africa (CANSA). Activists demand Roche drops cost of vital breast cancer medicine. Available online at: https://cansa.org.za/activists-demand-roche-drops-cost-of-vital-breast-cancer-medicine/ (Accessed March 11, 2026).

[84] Hakim D. Humira’s best-selling drug formula: start at a high price. Go higher. New York Times. (2018 January 6). Available online at: https://www.nytimes.com/2018/01/06/business/humira-drug-prices.html (Accessed February 17, 2026).

[85] Discovery Health. The challenge of funding cancer treatment. (2019 Jun). Available online at: https://www.discovery.co.za/corporate/funding-cancer-treatment (Accessed February 18, 2026).

[86] Gupta N Verma RK Gupta S Prinja S. Cost effectiveness of trastuzumab for management of breast cancer in India. JCO Glob Oncol. (2020) 6:205–16. 10.1200/JGO.19.00293 32045547 PMC7051799

[87] Gupta D Singh A Gupta N Mehra N Bahuguna P Aggarwal V. Cost-effectiveness of the first line treatment options for metastatic renal cell carcinoma in India. JCO Glob Oncol. (2023) 9:e2200246. 10.1200/GO.22.00246 36795991 PMC10166401

[88] INVC. Beyfortus immunization in Noida: price, dose, schedule and vaccination. (2026 March 12). Available online at: https://invc.co.in/rsv/beyfortus-immunization/noida (Accessed February 17, 2026).

[89] Nguyen AQ Tran OTM Nguyen PK Nguyen HT. Cost-effectiveness of one-year adjuvant trastuzumab therapy in treatment for early-stage breast cancer patients with HER2 + in Vietnam. PLoS One. (2024) 19(3):e0300474. 10.1371/journal.pone.0300474 38489305 PMC10942069

[90] VietnamNet Global. Cancer patients struggle with high treatment costs. (2026 February 9). Available online at: https://vietnamnet.vn/en/cancer-patients-struggle-with-high-treatment-costs-2488692.html (Accessed February 17, 2026).

[91] Wellcome Trust & International AIDS Vaccine Initiative (IAVI). (2020). Expanding access to monoclonal antibody-based products: A Global Call to Action. Available online at: https://cms.wellcome.org/sites/default/files/expanding-access-to-monoclonal-antibody-based-products.pdf (Accessed January 14, 2026).

[92] Wang Q Nader A Peppercorn A Skingsley A Lloyd E Stella AO. Clinical pharmacology approaches to predict efficacy of monoclonal antibodies against emerging SARS-CoV-2 variants. Clin Transl Sci. (2025) 18(12):e70421. 10.1111/cts.70421 41294924 PMC12649055

[93] Ovacik M Lin K. Tutorial on monoclonal antibody pharmacokinetics and its considerations in early development. Clin Transl Sci. (2018) 11(6):540–52. 10.1111/cts.12567 29877608 PMC6226118

[94] Mahajan A Dever J, Center for Global Health Policy and Politics. Pricing of Monoclonal Antibodies: The Need for Greater Transparency to Promote Globe. Available online at: https://ghpp.georgetown.edu/expert-column/pricing-of-monoclonal-antibodies-the-need-for-greater-transparency-to-promote-global-access/ (Accessed March 3, 2026).

[95] Otsubo R Yasui T. Monoclonal antibody therapeutics for infectious diseases: beyond normal human immunoglobulin. Pharmacol Ther. (2022) 240:108233. 10.1016/j.pharmthera.2022.108233 35738431 PMC9212443

[96] Tulchinsky TH. John snow, cholera, the broad street pump; waterborne diseases then and now. Case Stud Public Health. (2018) 1:77–99. 10.1016/B978-0-12-804571-8.00017-2

[97] George CM Monira S Sack DA Rashid MU Saif-Ur-Rahman KM Mahmud T. Randomized controlled trial of hospital-based hygiene and water treatment intervention (CHoBI7) to reduce cholera. Emerg Infect Dis. (2016) 22:233–41. 10.3201/eid2202.151175 26811968 PMC4734520

[98] Baranova DE Chen L Destrempes M Meade H Mantis NJ. Passive immunity to *Vibrio cholerae* O1 afforded by a human monoclonal IgA1 antibody expressed in milk. Pathog Immun. (2020) 5(1):89–116. 10.20411/pai.v5i1.370 34136728 PMC8204294

[99] Yongky A Xu J Tian J Oliveira C Zhao J McFarland K. Process intensification in fed-batch production bioreactors using non-perfusion seed cultures. MAbs. (2019) 11(8):1502–14. 10.1080/19420862.2019.1652075 31379298 PMC6816350

[100] Bielser JM Wolf M Souquet J Broly H Morbidelli M. Perfusion mammalian cell culture for recombinant protein manufacturing – a critical review. Biotechnol Adv. (2018) 36(5):1328–40. 10.1016/j.biotechadv.2018.04.011 29738813

[101] Gupta P Kateja N Mishra S Kaur H Rathore AS. Economic assessment of continuous processing for manufacturing of biotherapeutics. Biotechnol Prog. (2021) 37:e3108. 10.1002/btpr.3108 33305493

[102] DeFife K Pierce LN. Versatility of a single-use bioreactor platform for culture of diverse cell types. BioPharm Int. (2009) 22(2):30–7.

[103] Pierce LN Shabram P. Scalability of a disposable bioreactor from 25 L–500 L run in perfusion mode with a CHO-based cell line. BioProcess J. (2004) 3(4):51–6. 10.12665/J34.Pierce

[104] Africa CDC. Centers for Disease Control and Prevention. New Momentum for Monoclonal Antibodies Manufacturing in Africa. (9 May 2025). Available online at: https://africacdc.org/news-item/new-momentum-for-monoclonal-antibodies-manufacturing-in-africa/#:∼:text=News%20/%20Blog-,New%20Momentum%20for%20Monoclonal%20Antibodies%20Manufacturing%20in%20Africa,in%20R&D%20and%20uncertain%20markets (Accessed March 30, 2026).

[105] Angarita M Müller-Späth T Baur D Lievrouw R Lissens G Morbidelli M. Twin-column CaptureSMB: a novel cyclic process for protein A affinity chromatography. J Chromatogr A. (2015) 1389:85–95. 10.1016/j.chroma.2015.02.046 25748537

[106] Zhang S Chen W Zhou J Liang Q Zhang Y Su M. The benefits and safety of monoclonal antibodies: implications for cancer immunotherapy. J Inflamm Res. (2015) 18:4335–57. 10.2147/JIR.S499403 PMC11952073 40162076

[107] Grilo AL Mantalaris A. The increasingly human and profitable monoclonal antibody market. Trends Biotechnol. (2019) 37(1):9–16. 10.1016/j.tibtech.2018.05.014 29945725

[108] Atal I Trinquart L Ravaud P Porcher R. A mapping of 115,000 randomized trials revealed a mismatch between research effort and health needs in non–high-income regions. J Clin Epidemiol. (2018) 98:123–32. 10.1016/j.jclinepi.2018.01.006 29360559

[109] International AIDS Vaccine Initiative (IAVI), Impact Global Health. Access to Monoclonal Antibodies in Africa: A Call to Action. New York (NY): IAVI (2024). Available online at: https://www.iavi.org/wp-content/uploads/2024/12/Access-to-monoclonal-antibodies-in-Africa.pdf (Accessed January 2, 2026).

[110] Chung C Kudchodkar SB Chung CN Park YK Xu Z Pardi N. Expanding the reach of monoclonal antibodies: a review of synthetic nucleic acid delivery in immunotherapy. Antibodies. (2023) 12(3):46. 10.3390/antib12030046 37489368 PMC10366852

[111] SU Support. The most overlooked costs in monoclonal antibody production. Single Use Support. Available online at: https://www.susupport.com/blogs/biopharmaceutical-products/most-overlooked-costs-monoclonal-antibody-production (Accessed March 2, 2026).

[112] Ötes O Bernhardt C Brandt K Flato H Klingler O Landrock K. Moving to CoPACaPAnA: implementation of a continuous protein A capture process for antibody applications within an end-to-end single-use GMP manufacturing downstream process. Biotechnol Rep (Amst). (2020) 26:e00465. 10.1016/j.btre.2020.e00465 PMC7218147 32420053

[113] Kikuchi T Ohira S Yamaguchi H. Viable cell density as an indicator for dynamic feeding strategy in fed-batch and perfusion cho cell culture. Sci Rep. (2025) 15(1). 10.1038/s41598-025-28316-8. PMC12749995 41462110

[114] Lin H Leighty RW Godfrey S Wang SB. Principles and approach to developing mammalian cell culture media for high cell density perfusion process leveraging established fed-batch media. Biotechnol Prog. (2017) 33(4):891–901. 10.1002/btpr.2472 28371394

[115] Pollard D Brower M Richardson D. Progress toward automated single-use continuous monoclonal antibody manufacturing via the protein refinery operations lab. Contin Biomanuf Innovative Technol Methods. (2017) 1:107–30. 10.1002/9783527699902.ch4

[116] World Health Organization. Respiratory syncytial virus (RSV). (2025 December 19). Available online at: https://www.who.int/news-room/fact-sheets/detail/respiratory-syncytial-virus-(rsv) (Accessed January 11, 2026).

[117] Li Y Wang X Blau DM Caballero MT Feikin DR Gill CJ. Global, regional, and national disease burden estimates of acute lower respiratory infections due to respiratory syncytial virus in children younger than 5 years in 2019: a systematic analysis. Lancet. (2022) 399(10340):2047–64. 10.1016/S0140-6736(22)00478-0 35598608 PMC7613574

