# Supplementary material for: Developing affordable monoclonal antibodies for LMICs through high performance manufacturing processes and LMIC based commercial manufacturing
Source: Front Pediatr. 2026 Sep 14;14:1880830. doi: 10.3389/fped.2026.1880830 (PMC13616801; doi:10.3389/fped.2026.1880830)
Supplement: Supplementary file 1 [file Supplementaryfile1.docx]

**Supplementary Table 1: Residual DNA Study – Post Protein A**

A260/A280 ratio measurements of known samples compared against in process ZAC3 samples Pre- and Post- Protein A column purification. Plasmid and BSA are used as controls, followed by increasing DNA additions to show the effects of increasing residual DNA on A260/280 ratios.

| Residual DNA Study - Post Protein A | | | | | |
| --- | --- | --- | --- | --- | --- |
| Sample | ng/ul (DNA) | A260 | A280 | A260/A280 | Constant |
| Pure Plasmid ~450 ng/ml DNA | 444.00 | 8.88 | 4.834 | 1.84 | 50.00 |
| Pure Plasmid ~450 ng/ml DNA | 447.93 | 8.959 | 4.856 | 1.84 | 50.00 |
| 1.0 mg/ml BSA Standard |  | 0.493 | 0.568 | 0.87 | 50.00 |
| 1.0 mg/ml BSA Standard |  | 0.518 | 0.624 | 0.83 | 50.00 |
| 2.0 mg/ml BSA Standard |  | 0.838 | 1.166 | 0.72 | 50.00 |
| 2.0 mg/ml BSA Standard |  | 0.848 | 1.14 | 0.74 | 50.00 |
| 1.0 mg/ml BSA + 50 ng/ul DNA |  | 1.294 | 0.998 | 1.30 | 50.00 |
| 1.0 mg/ml BSA + 50 ng/ul DNA |  | 1.27 | 0.913 | 1.39 | 50.00 |
| 2.0 mg/ml BSA + 50 ng/ul DNA |  | 1.552 | 1.455 | 1.07 | 50.00 |
| 2.0 mg/ml BSA + 50 ng/ul DNA |  | 1.582 | 1.485 | 1.07 | 50.00 |
| Zac3 Elution + 50 ng/ml DNA |  | 9.796 | 13.003 | 0.75 | 50.00 |
| Zac3 Elution + 50 ng/ml DNA |  | 9.952 | 13.244 | 0.75 | 50.00 |
| Zac3 Elution + 100 ng/ml DNA |  | 9.478 | 10.654 | 0.89 | 50.00 |
| Zac3 Elution + 100 ng/ml DNA |  | 9.533 | 12.223 | 0.78 | 50.00 |
| Zac3 Elution + 250 ng/ml DNA |  | 6.739 | 7.8 | 0.86 | 50.00 |
| Zac3 Elution + 250 ng/ml DNA |  | 7.071 | 8.364 | 0.85 | 50.00 |
| Zac3 Harvest/Protein A Load |  | 42.647 | 46.3 | 0.92 | 50.00 |
| Zac3 Harvest/Protein A Load |  | 42.821 | 46.219 | 0.93 | 50.00 |
| Zac3 Protein A Elution |  | 6.815 | 12.594 | 0.54 | 50.00 |
| Zac3 Protein A Elution |  | 6.963 | 12.69 | 0.55 | 50.00 |
| Pure DNA Ratio ≈ 1.8 |  | | | | |
| Pure Protein Ratio = 0.5-0.7 |  |  |  |  |  |
| Sample containing DNA and Protein |  |  |  |  |  |

**Supplementary Figure 1: ProA HCP Removal Gel**

4-12% Tris-Glycine Gel of Protein A purification column steps, and their associated samples for ZAC3 affinity purification. Lower banding is attributed to HCP, which is resolved and shown to be removed at elution.

**Supplementary Figure 2: CEX HMW Aggregate Removal Gel**

4-12% Tris-Glycine Gel of ZAC3 Cationic Exchange Chromatography. ZAC3 antibody was processed under bind and elute conditions. CEX column strip shows HMW aggregate removal, as well as some residual HCP’s.
